# Building an Antibiotic Stewardship Program: An Interactive Teaching Module for Medical Students

**DOI:** 10.15766/mep_2374-8265.10726

**Published:** 2018-06-26

**Authors:** Jennifer L. Hsu

**Affiliations:** 1 Associate Professor, Department of Internal Medicine, University of South Dakota, Sanford School of Medicine

**Keywords:** Infection, Antibiotics, Antibiotic Resistance, Antimicrobial Stewardship, Antibiotic Stewardship

## Abstract

**Introduction:**

Given the increasing importance of antibiotic stewardship, an understanding of the basic tenets of antibiotic prescribing is critical for all medical students. This engaged learning module focuses on terminology and foundational concepts in antibiotic stewardship while assisting learners with application in their early clinical practice.

**Methods:**

This module was developed for third-year internal medicine clerkship students at the University of South Dakota, Sanford School of Medicine. The students participated in an introductory discussion of the harms of antibiotic overuse in a large-group format, followed by small-group work to develop a miniature antibiotic stewardship program. Upon completion of the small-group portion, the large group reconvened to share antibiotic stewardship strategies and to complete the session with additional training regarding individual provider and system-level antibiotic stewardship strategies.

**Results:**

Approximately 150 students have participated in this module. Through use of the module, students have been highly engaged in identifying antibiotic stewardship interventions in their early practice and creating potential solutions. Themes identified during analysis of one-minute paper responses demonstrated student learning around the rationale for antibiotic stewardship programs, implementation strategies, and patient education.

**Discussion:**

This module was primarily developed for medical students, but it could easily be adapted for use with learners of varying levels. Additionally, it could be implemented as an interprofessional exercise to leverage the expertise of trainees in pharmacy and nursing.

## Educational Objectives

By the end of this activity, learners will be able to:

1. Define the term *antibiotic stewardship.*
2. List the benefits of antibiotic stewardship.
3. Describe at least three evidence-based strategies for implementation of antibiotic stewardship.
4. Design a focused antibiotic stewardship intervention relevant to their clinic experience.

## Introduction

Multiple harmful effects of inappropriate antibiotic use have been identified, including emergence of antibiotic-resistant pathogens, increased health care costs, and heightened risk for adverse drug events. These issues directly affect patients and health systems, as well as society as a whole, and they are magnified in an era of slowed antibiotic development. The concept of antibiotic stewardship was developed in the mid-1990s in response to these threats.^1^ More recently, antibiotic stewardship has come to represent structured programs aimed at improving the utilization and monitoring of antibiotics.^2^ The National Action Plan for Combating Antibiotic-Resistant Bacteria, issued by the White House in 2015, reinforced the importance of antibiotic stewardship programs (ASPs) by requiring them in all acute care hospitals by 2020.^3^

While there are many facets of an effective ASP, education is a cornerstone of most programs.^4,5^ Education has traditionally been focused on clinicians and clinical pharmacists. Little attention has been paid to trainees, including medical students and residents, who may benefit more from ASP education as they are early in their development of prescribing habits.^5,6^ In the preclinical years, 66% of institutions surveyed in one study reported integration of ASP concepts in their preclinical microbiology curricula.^7^ A second study showed that 90% of graduating medical students responded that they would like more education on the appropriate use of antibiotics.^6^ Additionally, a study involving 48 internal medicine residents revealed that they had primary responsibility for initiation of broad-spectrum antibiotics.^8^ Taken together, these findings show that the opportunity to reach trainees at critical moments in their careers may lead to enhanced antibiotic stewardship over the duration of their careers.

Given the gap in ASP education highlighted above, educational activities and curricula are increasingly important.^9,10^ Passive education techniques are useful for increased provider knowledge, whereas active education associated with specific patient cases or prescribing data has increased influence on prescribing behavior and is longer lived.^5^ In *MedEdPORTAL,* this approach has been undertaken using simulation to teach ASP concepts to infectious disease fellows, but limited curricula are available for undergraduate medical students.^11^ The module presented here aims to use engaged learning techniques to guide medical students through development of a mock ASP intervention relevant to their personal clinical experiences. This module was initially developed to introduce family medicine residents to ASP concepts. It has been adapted and used annually since 2015 for third-year internal medicine clerkship students.

## Methods

I designed this teaching module to develop a foundation of ASP knowledge in a single didactic session for inclusion within the time constraints of a busy third-year internal medicine clerkship at the University of South Dakota, Sanford School of Medicine. Addition of this content within the middle portion of the third-year clerkship allowed students to draw on their clinical encounters during the interactive portion of the session. All students participated in didactic sessions covering principles of quality improvement and patient safety prior to engagement in this module. Given the varied learner experiences with ASP, students reviewed an article about general principles of antibiotic stewardship in preparation for the session.^2^ Facilitators actively practicing in ASP programs provided guidance and real-world examples during the discussion.

### Learning Environment

I developed this module for use in a class of 30 students, which then divided into groups of three to five students for a small-group activity. I presented the session in a classroom equipped with a computer and projector, and students received handouts to guide them through the small-group work.

### Teaching Plan

The teaching module presented included the following: PowerPoint slide set with instructor notes (Appendix A), student worksheet entitled “Building an Antibiotic Stewardship Program” (Appendix B), and one-minute paper evaluation (Appendix C).^2,11^ Students began the session in the large group, brainstorming costs and concerns associated with inappropriate antibiotic use. This discussion set the stage for a brief slide-based lecture on the harms of inappropriate antibiotic use and the rationale behind antibiotic stewardship. Small groups of three to five students worked through the “Building an Antibiotic Stewardship Program” worksheet. With this activity, students considered personal clinical experiences and opportunities for implementation of antibiotic stewardship principles. With these experiences in mind, they outlined a focused antibiotic stewardship intervention or program and developed one specific goal and one to three objectives for their program. They answered questions about program logistics, including who, where, and how, as well as identifying necessary resources (funding, staff, etc.) and potential barriers. Lastly, they created outcome measures. After completion of the small-group activity, students returned to the larger group for discussion of their ASPs. Students shared suggestions for enhancement of ASPs, as well as opportunities in their practices for implementation of ASP principles. The session wrapped up with a brief slide-based lecture to reinforce key ASP strategies. The session lasted approximately 75 minutes (see the Table).

**Table.** Teaching Plan With Time Estimates

Minutes Allotted	Activity	Group Size	Purpose of Activity
Pre	Read review article about ASPs.	Large	Background knowledge.
10	Introduction: brainstormed adverse effects of inappropriate antibiotic use and presented slides with data on outcomes of inappropriate antibiotic use (Slides 1–17).	Large	Reviewed information from the prereading assignment, which established the importance of ASPs.
20	Building an ASP: utilizing the worksheet, students worked together to select a focused ASP intervention and discuss the goal, objectives, logistics of implementation, potential barriers, and outcome assessment (Slide 18).	Small	Students used their clinical experiences to identify practical opportunities for ASP implementation, thus applying the information they learned in the prereading and first portion of this teaching module.
20	Presented and discussed small-group ASP proposals.	Large	Students shared their proposals with the large group, which provoked questions, clarifications, and refinement of the small-group ideas.
20	Presented slides with key ASP principles (Slides 20–37).	Large	Reinforced the take-home points and oriented students to local ASP resources if available.
5	Wrap-up: questions and one-minute paper completion.	Large	Provided a brief period for clarification of concepts and completion of an evaluation.

Abbreviation: ASP, antibiotic stewardship program.

### Evaluation

At the end of the session, students highlighted key concepts they had learned and outstanding questions using a one-minute paper (Appendix C).^12^ This activity confirmed that that the learners had accomplished Educational Objectives 1–3. Students also shared an ASP strategy they wished to implement in their practice, which solidified Educational Objective 4. After the session, I emailed students to clarify unclear concepts from the one-minute paper. I conducted a thematic analysis of the results from the data collection.

## Results

This interactive workshop has been successfully implemented annually for three classes of third-year internal medicine clerks. The initial presentation was scaled for a class of 40 students; however, more recently, it has been successfully utilized in a class of 55 students, including students participating through videoconferencing at remote sites. Approximately 150 students have participated in the module (45–55 annually), and in general, antibiotic stewardship has been a new topic for them. Students' ideas for antibiotic stewardship interventions have varied widely but have generally centered on stewardship opportunities witnessed during clinical rotations. Student engagement has been uniformly high throughout the sessions. Specific comments included the following: “Audience participation was a great way to hear multiple perspectives on the same problems,” and “This session provided realistic ways to approach antibiotic prescribing and stewardship when it feels like an overwhelming and insurmountable problem.” The one-minute paper response rate averaged 37%. Analysis of the one-minute paper (Appendix C) responses revealed several themes, as shown below.

### Concept Learned

• Importance of antibiotic stewardship:
  ○ “Antibiotic stewardship is everyone's responsibility.”
  ○ “ASP is becoming much more relevant.”
  ○ “This gives me a new outlook on antibiotic overprescribing.”
• ASP concepts and implementation methods:
  ○ “Importance of appropriate use criteria.”
  ○ “Implementing antibiotic cycling.”
  ○ “I had no idea how ASP could be used on a daily basis.”
• Safety-net antibiotic prescriptions (SNAP):
  ○ “The SNAP approach is a good idea to decrease antibiotic use.”
  ○ “How I could implement SNAP in my primary care clinic.”
• Systematic approach to antibiotic prescribing:
  ○ “Reassess empiric antibiotics after 48–72 hours.”
  ○ “Make sure the chosen antibiotic is active at the site of infection, not just against the bug.”
  ○ “Some drugs are no better when given IV vs. PO.”
  ○ “I should have a routine way to think about antibiotics: diagnosis → pathogen → antibiotic.”

### Concept Unclear

• Practical implementation of ASP concepts:
  ○ “How do you do prospective audits?”
  ○ “How are ASPs integrated with EMRs?”
  ○ “How do you balance ASP with physician autonomy?”
• Evaluation of ASP program outcomes:
  ○ “How do you measure programmatic successes?”
  ○ “How do hospitals track antibiotic usage data?”
  ○ “Are outcomes actually different with stewardship? It seems difficult to measure even though logically we know what is needed.”
• Utilization of antibiograms:
  ○ “Does everyone have antibiograms?”
  ○ “Where can I locate antibiograms?”

### Concept to Try

• Systematic approach to antibiotic prescribing:
  ○ “Try nonantibiotic approaches first for viral syndromes.”
  ○ “Always use a systematic approach to antibiotic selection.”
  ○ “Ask questions of attendings to understand why they selected a specific antibiotic.”
  ○ “Consider antibiotics with narrowest spectrum possible.”
• Antibiograms and SNAP:
  ○ “Use antibiogram and ASP resources embedded in the EMR.”
  ○ “I'm going to suggest SNAP in my primary care clinic.”
• Patient education:
  ○ “I'm going to talk with patients more about why an antibiotic is not indicated.”
  ○ “I'm going to give patients resources for nonantibiotic treatments.”
  ○ “Engage my attending to find ways I can help with patient education, especially when they are pressed for time.”

## Discussion

The idea for this workshop was spurred by a faculty development conference on interactive learning. The workshop began as a team-based learning exercise, but over time, it was easier to guide students through a less structured format. Students have been highly engaged and appear to benefit from the free-form discussion that allows incorporation of meaningful real-life examples. As presented, this workshop is a single-session module incorporating antibiotic stewardship concepts in a busy third-year clerkship schedule. Implementation during the third-year clerkship engages students early in a time when they are beginning to envision themselves as clinical decision makers with opportunities to implement the presented information. The workshop has been successful with a group of resident learners as well. It is flexible and can be adapted to varying group sizes, levels of learners (clerkship, residency, and CME), and evolving content as the science behind ASP further develops.

### Limitations

Due to the need for a single, concise session, the primary limitation of this resource is the lack of objective outcome measures. Ideally, students would participate in a pre- and postworkshop assessment of their knowledge, as well as a longer-term assessment of their planned implementation of ASP strategies. For example, a follow-up survey during the fourth year would be very useful for assessing sustainability of the impact. This session provides only an introductory analysis of a topic that could certainly be expanded into a larger curriculum.

### Potential Future Directions

Evaluations from each workshop have been utilized to refine the presented content each year. The most recent cohort of student participants suggested a longer session to allow for more time in the large group discussing the small-group ASP proposals. They also requested inclusion of information regarding local ASP interventions and associated outcome data. In addition to adjustment of content, the workshop could easily be developed into an interprofessional exercise, engaging pharmacy, physician assistant, and nursing learners and enhancing the focus on patient safety and quality improvement.

Overall, antibiotic stewardship is an increasingly important topic for learners at varying levels. This concise module introduces terminology and basic concepts as a foundation for participants' further growth as antibiotic prescribers. When the material has been presented in an engaged learning format, learners have provided positive feedback on the experience.

## Appendices

A. ASP Presentation Slides.pptx
B. Building an ASP Worksheet.docx
C. One-Minute Paper.docx All appendices are peer reviewed as integral parts of the Original Publication.
