# Supplementary material for: Building an Antibiotic Stewardship Program: An Interactive Teaching Module for Medical Students
Source: MedEdPORTAL. 2018 Jun 26;14:10726. doi: 10.15766/mep_2374-8265.10726 (PMC6342413; doi:10.15766/mep_2374-8265.10726)
Supplement: Supplementary file 1 — A. ASP Presentation Slides.pptx B. Building an ASP Worksheet.docx C. One-Minute Paper.docx [file mep-14-10726-s001.zip › A._ASP_Presentation_Slides.pptx]

## Slide 1
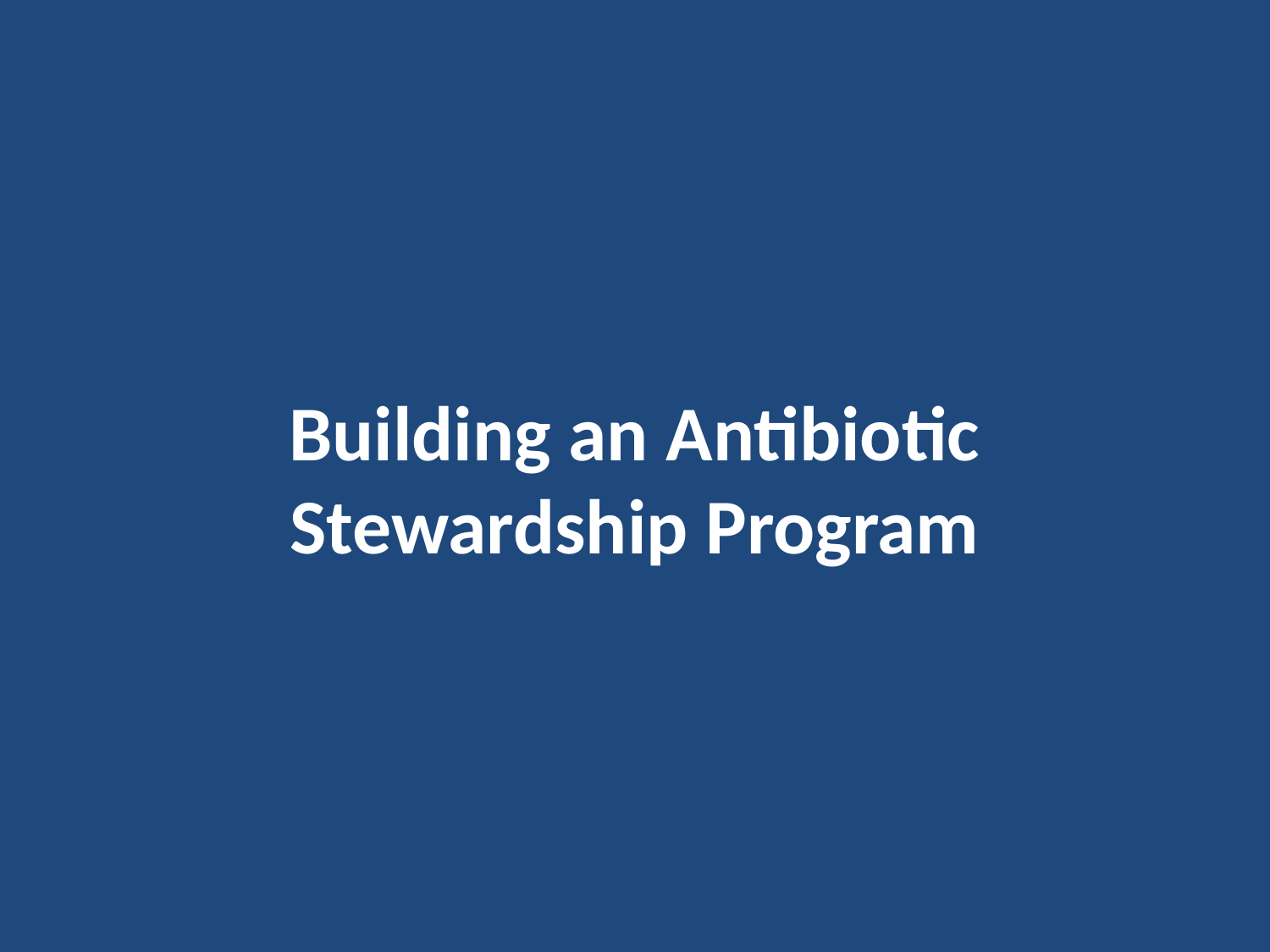

# Building an Antibiotic Stewardship Program

## Slide 2
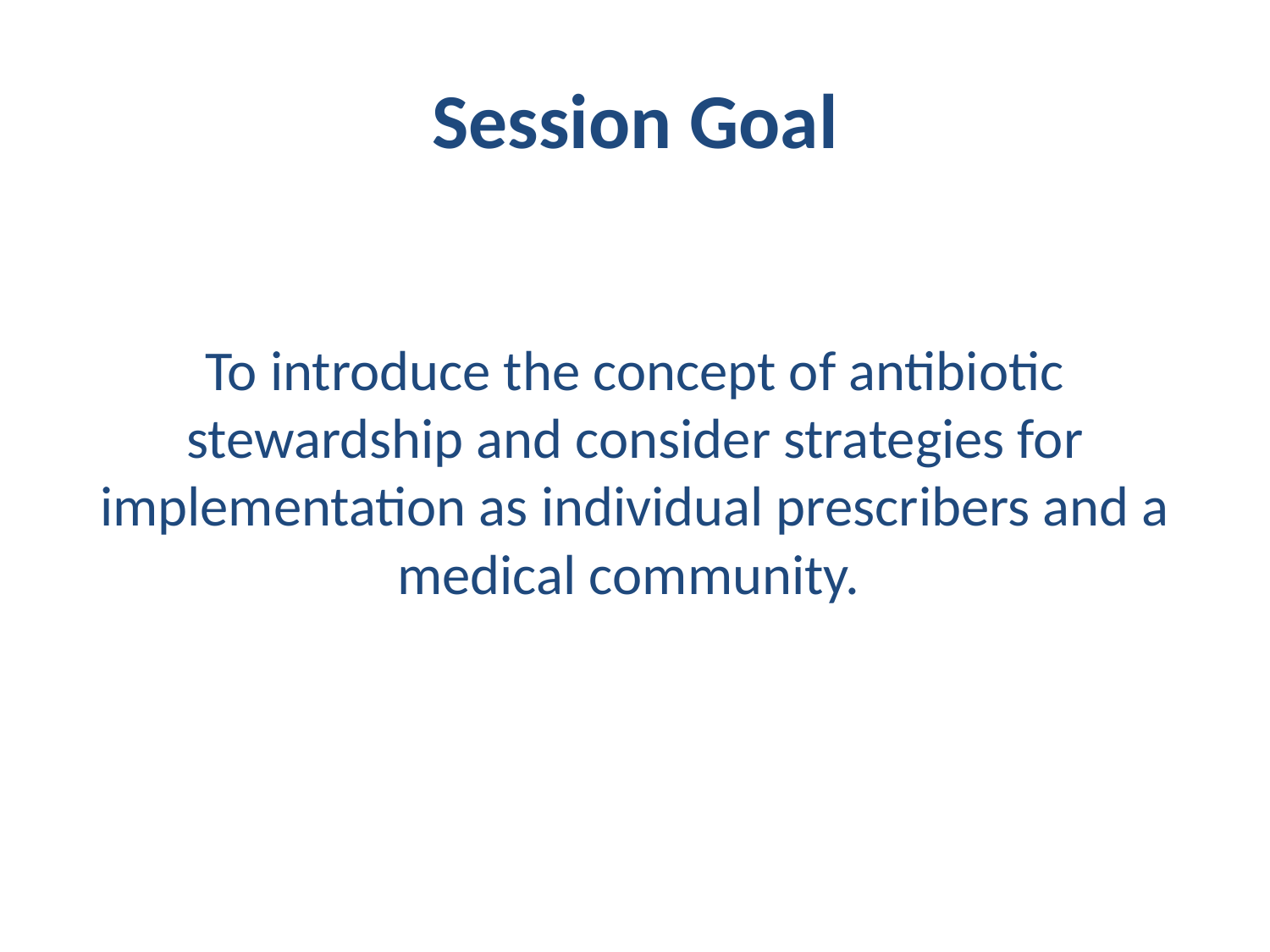

# Session Goal
To introduce the concept of antibiotic stewardship and consider strategies for implementation as individual prescribers and a medical community.

## Slide 3
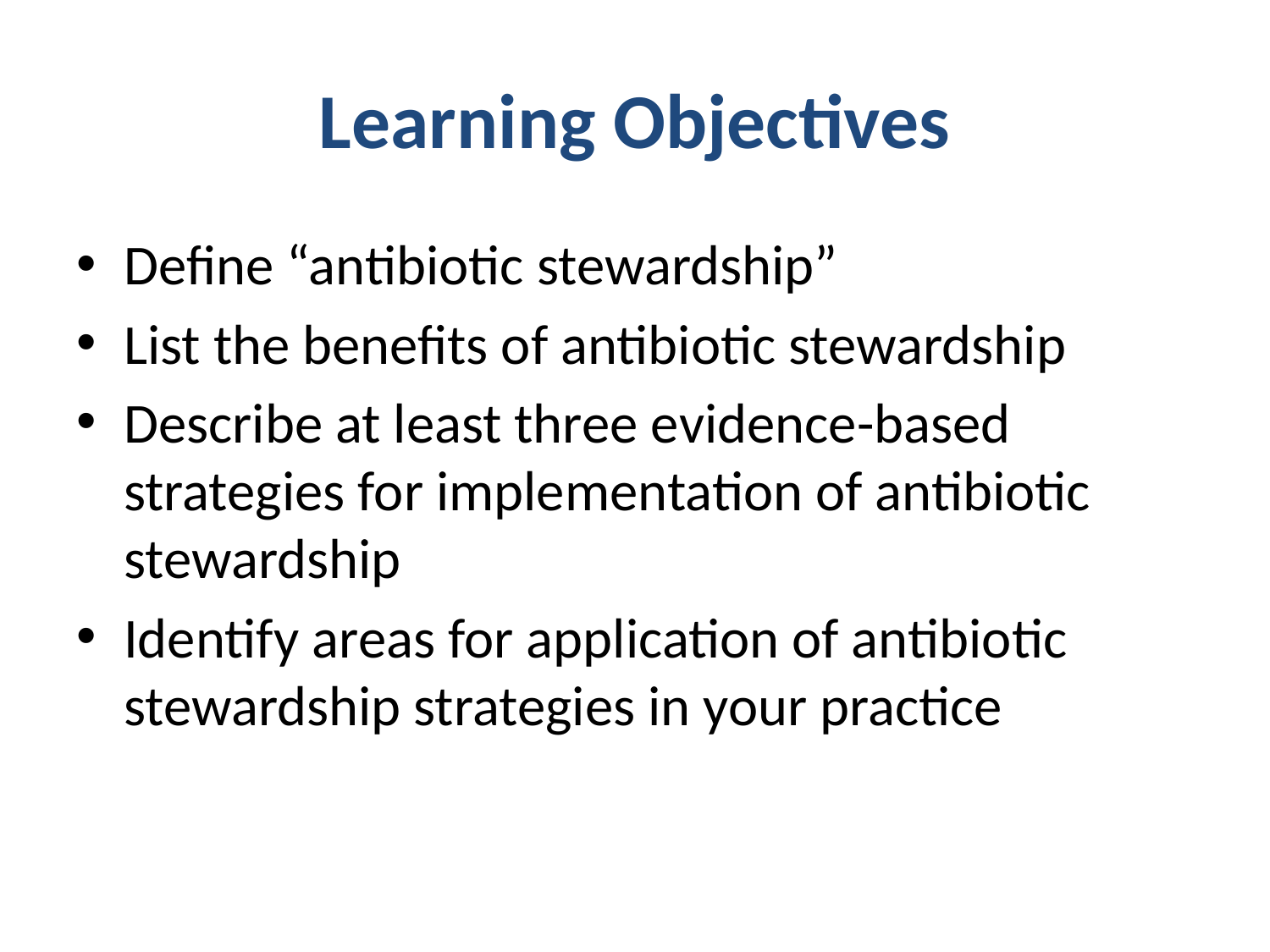

# Learning Objectives
Define “antibiotic stewardship”
List the benefits of antibiotic stewardship
Describe at least three evidence-based strategies for implementation of antibiotic stewardship
Identify areas for application of antibiotic stewardship strategies in your practice

## Slide 4
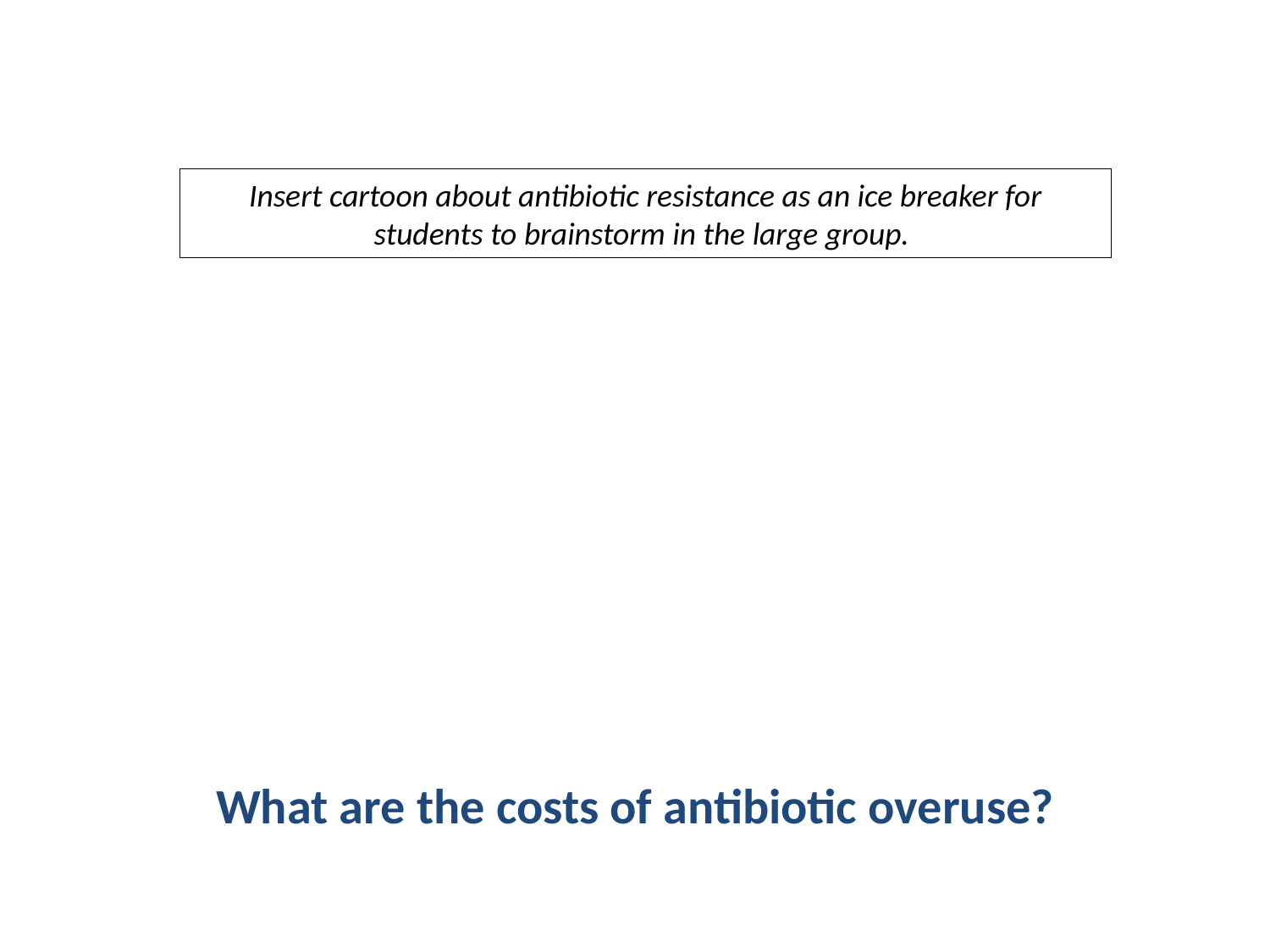

Insert cartoon about antibiotic resistance as an ice breaker for students to brainstorm in the large group.
# What are the costs of antibiotic overuse?

## Slide 5
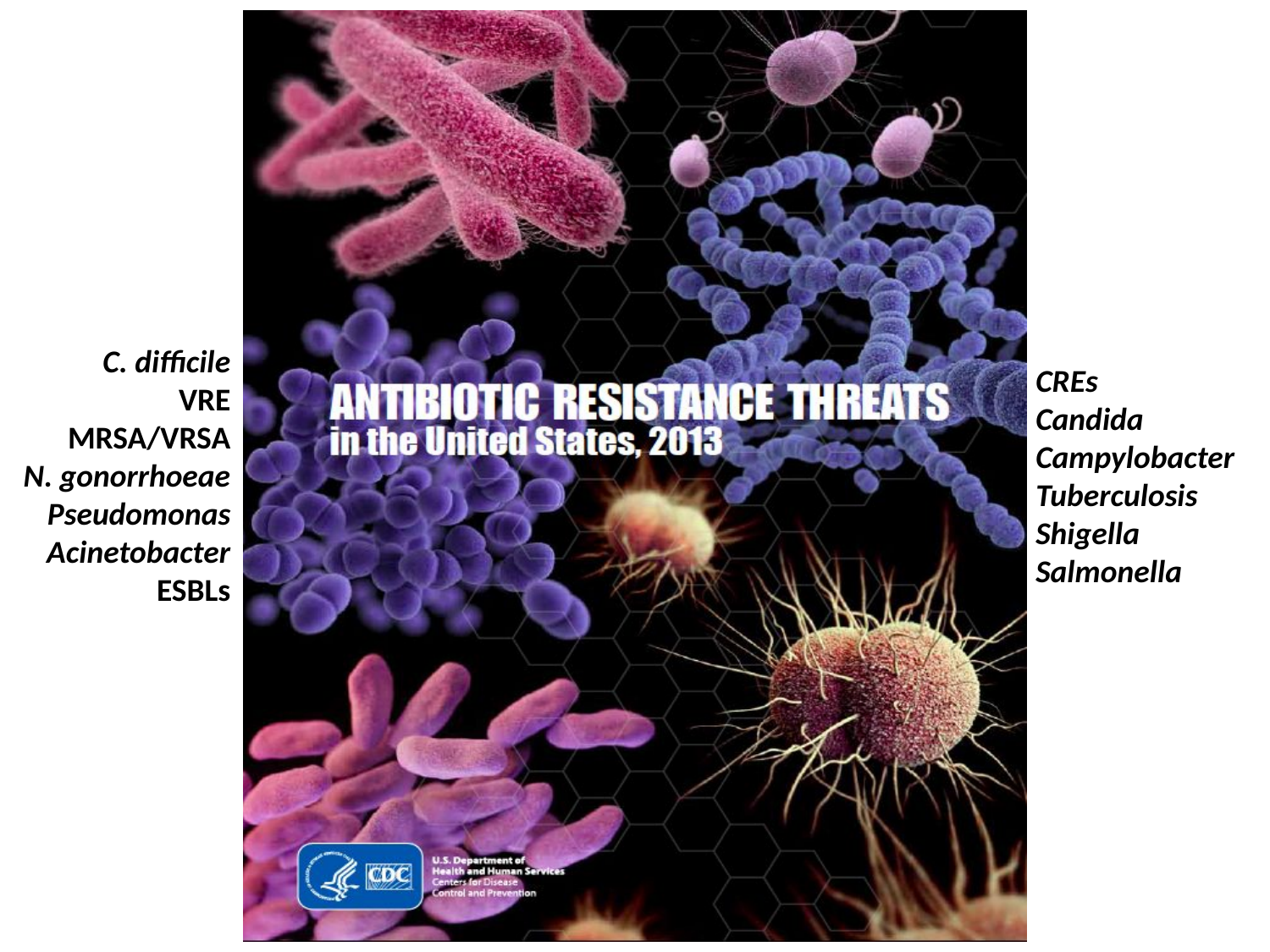

C. difficile
VRE
MRSA/VRSA
N. gonorrhoeae
Pseudomonas
Acinetobacter
ESBLs
CREs
Candida
Campylobacter
Tuberculosis
Shigella
Salmonella

## Slide 6
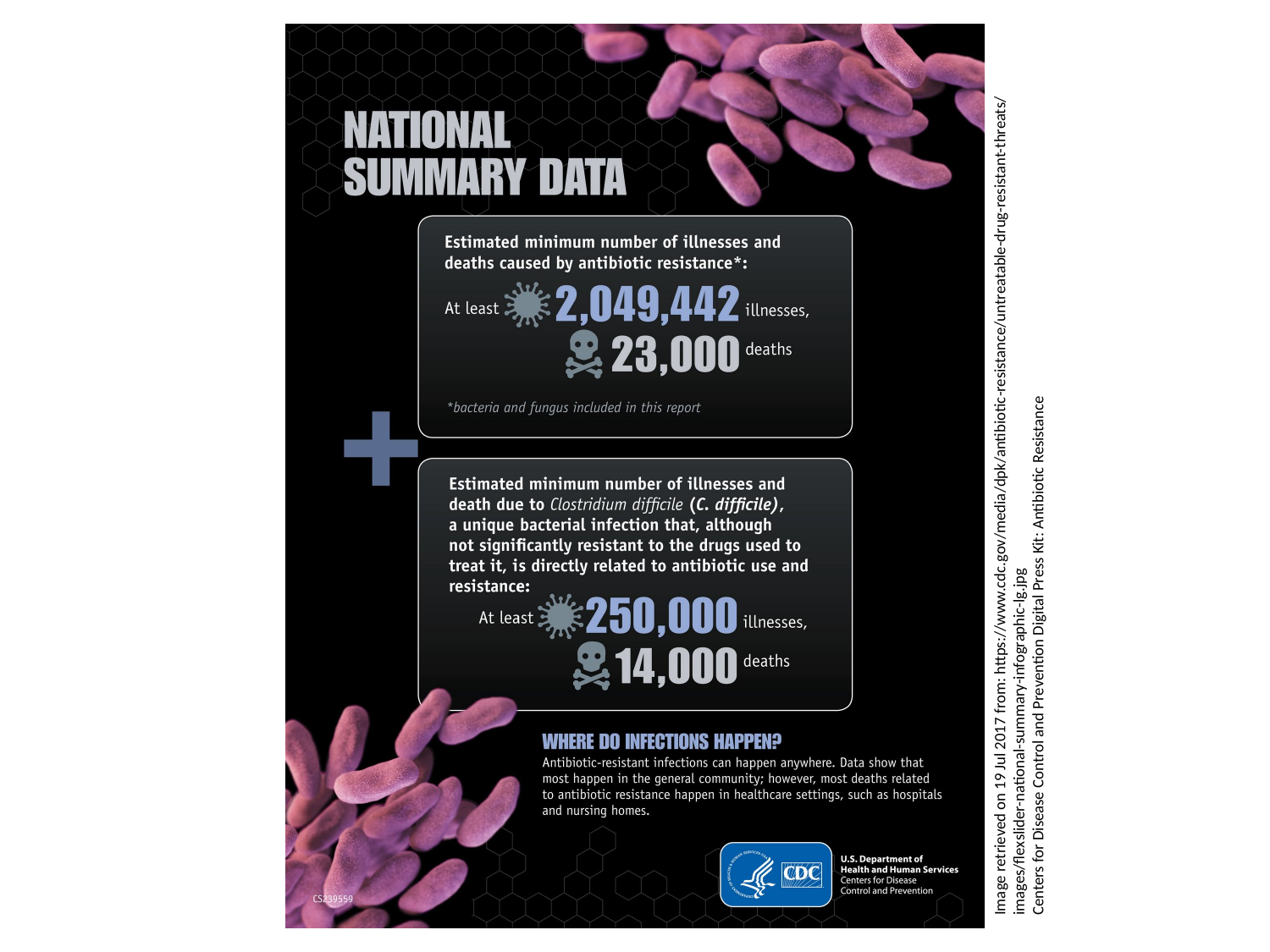

Image retrieved on 19 Jul 2017 from: https://www.cdc.gov/media/dpk/antibiotic-resistance/untreatable-drug-resistant-threats/images/flexslider-national-summary-infographic-lg.jpg
Centers for Disease Control and Prevention Digital Press Kit: Antibiotic Resistance

## Slide 7
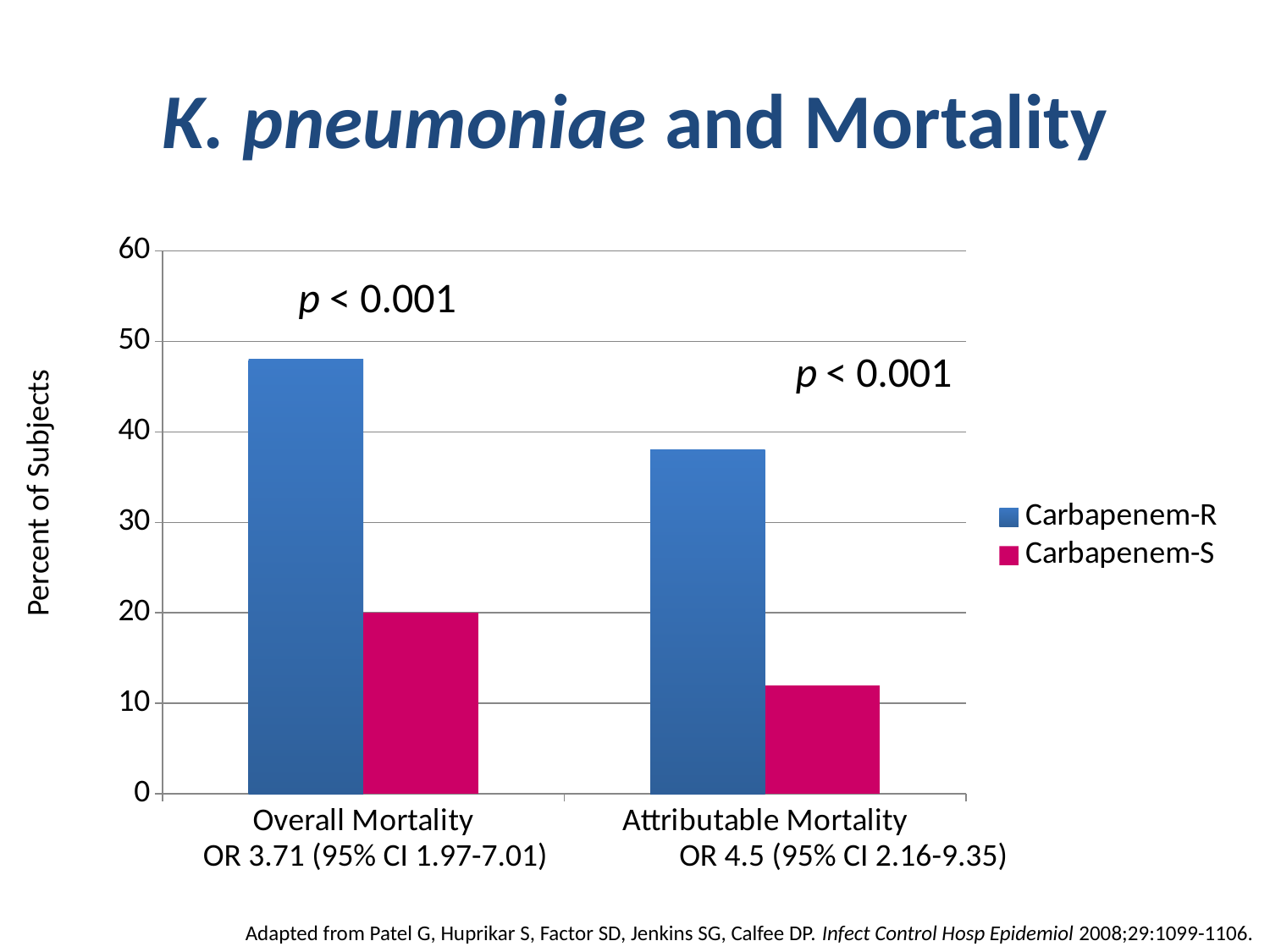

# K. pneumoniae and Mortality
### Chart
| Category | Carbapenem-R | Carbapenem-S |
|---|---|---|
| Overall Mortality | 48.0 | 20.0 |
| Attributable Mortality | 38.0 | 12.0 |p < 0.001
Percent of Subjects
OR 3.71 (95% CI 1.97-7.01)
OR 4.5 (95% CI 2.16-9.35)
Adapted from Patel G, Huprikar S, Factor SD, Jenkins SG, Calfee DP. Infect Control Hosp Epidemiol 2008;29:1099-1106.

## Slide 8
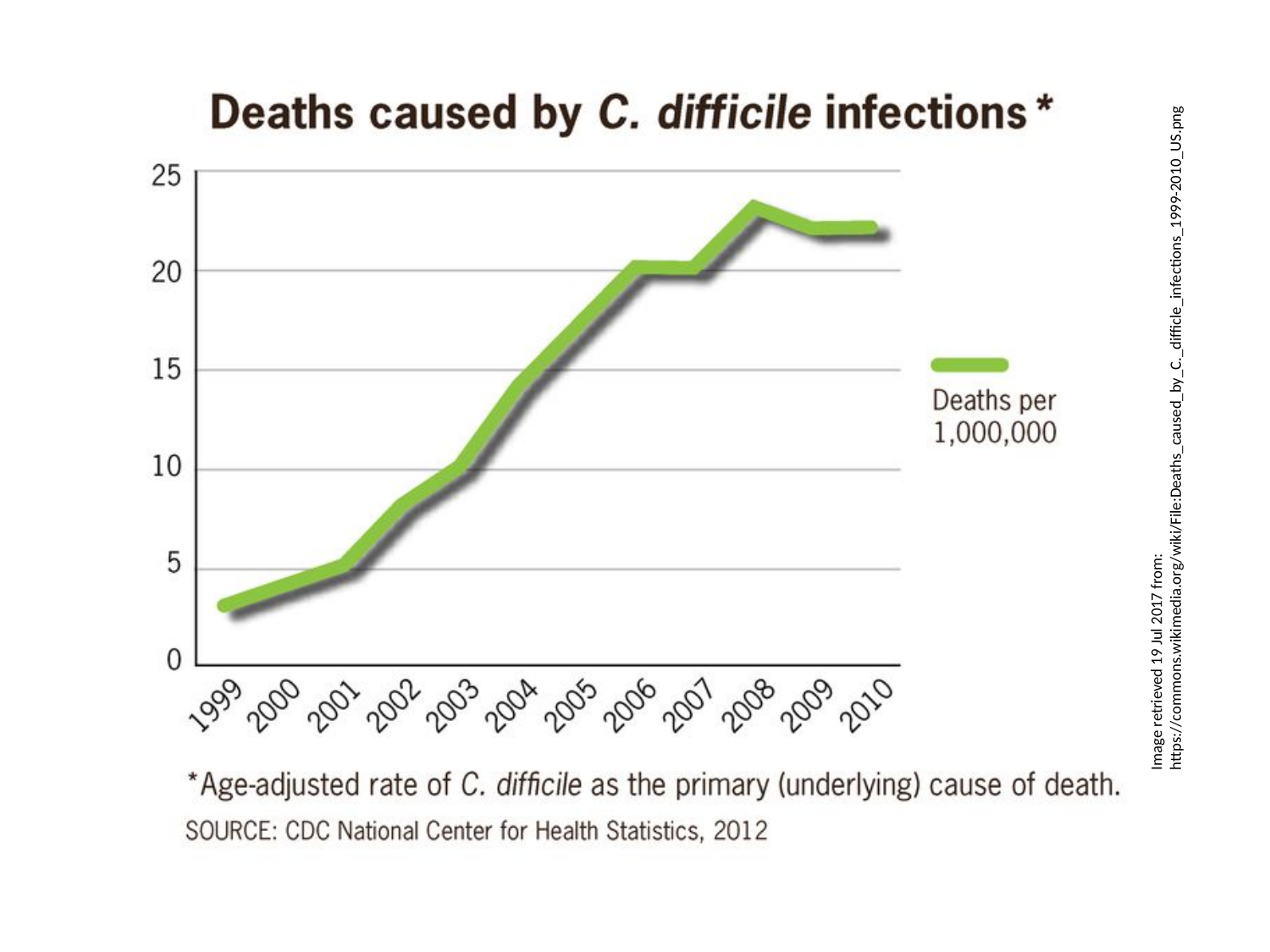

Image retrieved 19 Jul 2017 from: https://commons.wikimedia.org/wiki/File:Deaths_caused_by_C._difficle_infections_1999-2010_US.png

## Slide 9
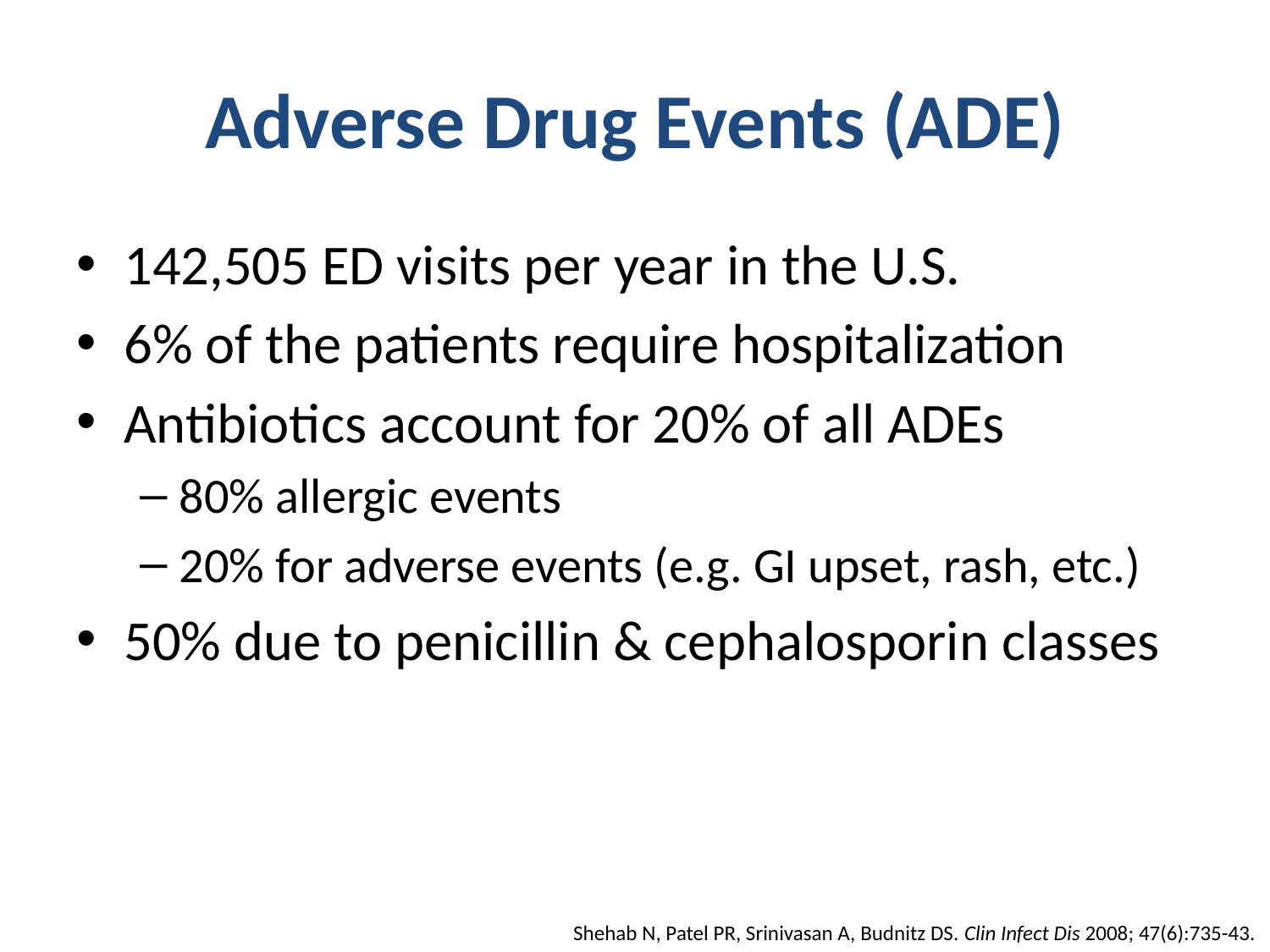

# Adverse Drug Events (ADE)
142,505 ED visits per year in the U.S.
6% of the patients require hospitalization
Antibiotics account for 20% of all ADEs
80% allergic events
20% for adverse events (e.g. GI upset, rash, etc.)
50% due to penicillin & cephalosporin classes
Shehab N, Patel PR, Srinivasan A, Budnitz DS. Clin Infect Dis 2008; 47(6):735-43.

## Slide 10
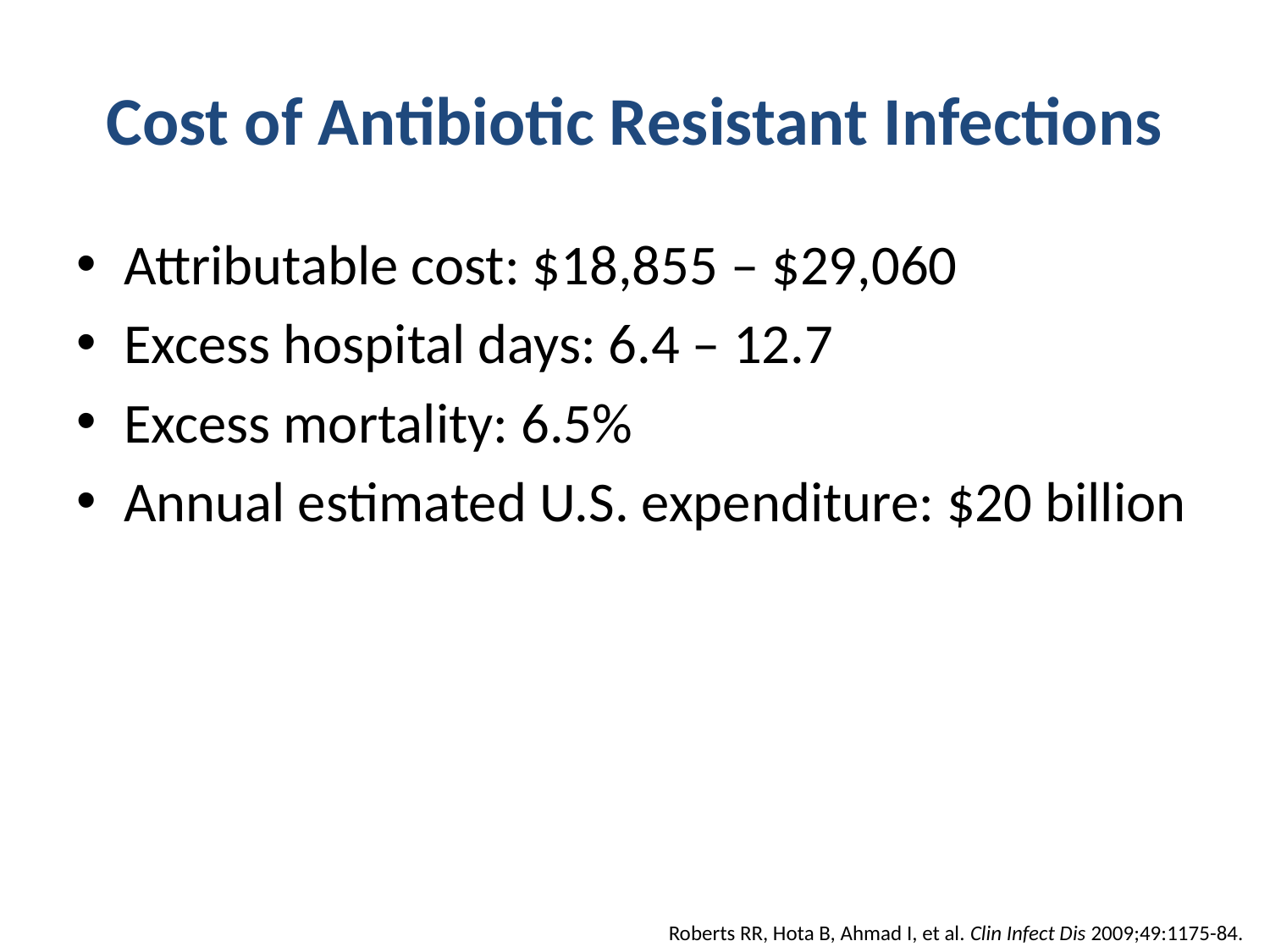

# Cost of Antibiotic Resistant Infections
Attributable cost: $18,855 – $29,060
Excess hospital days: 6.4 – 12.7
Excess mortality: 6.5%
Annual estimated U.S. expenditure: $20 billion
Roberts RR, Hota B, Ahmad I, et al. Clin Infect Dis 2009;49:1175-84.

## Slide 11
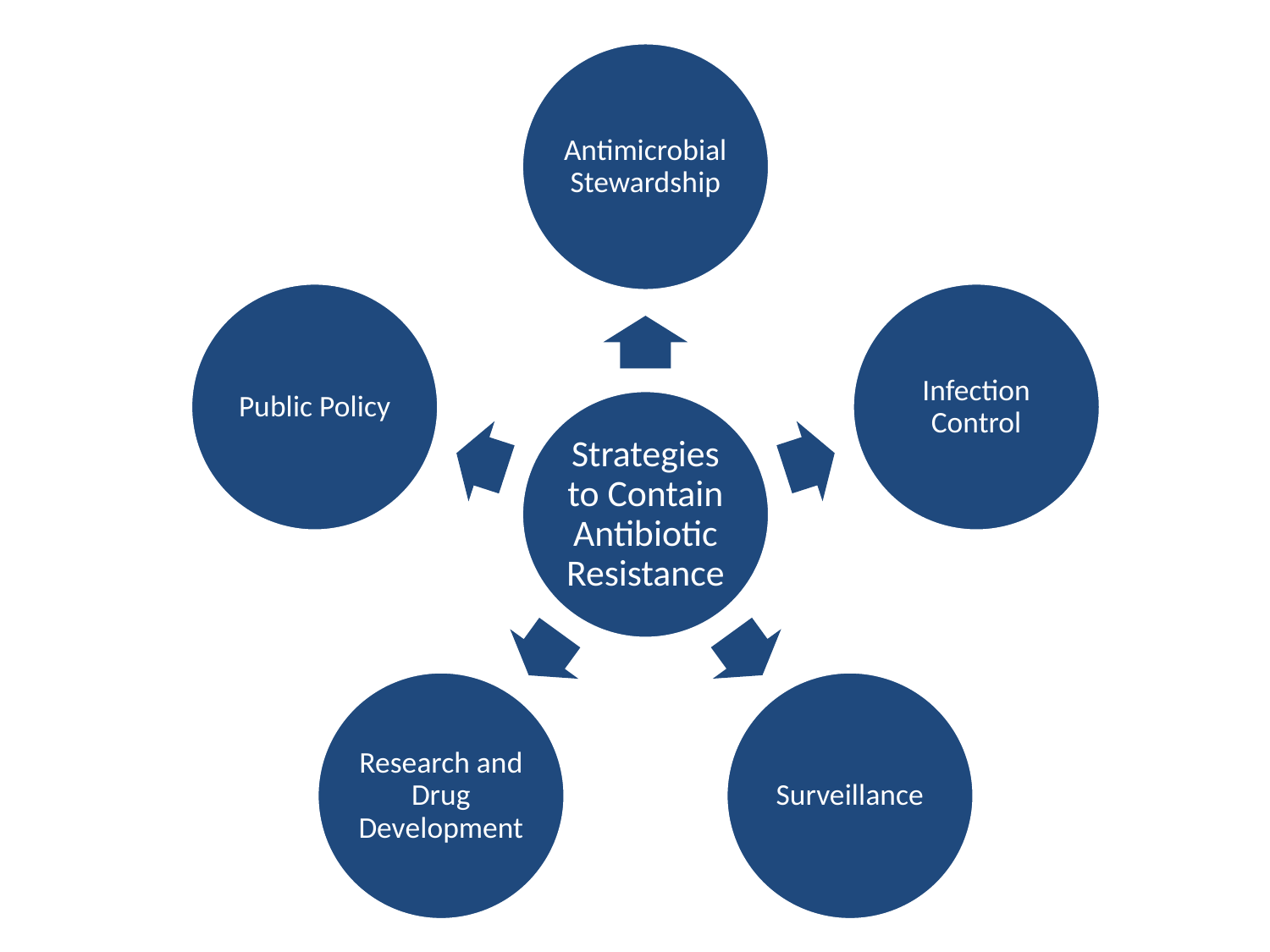

## Slide 12
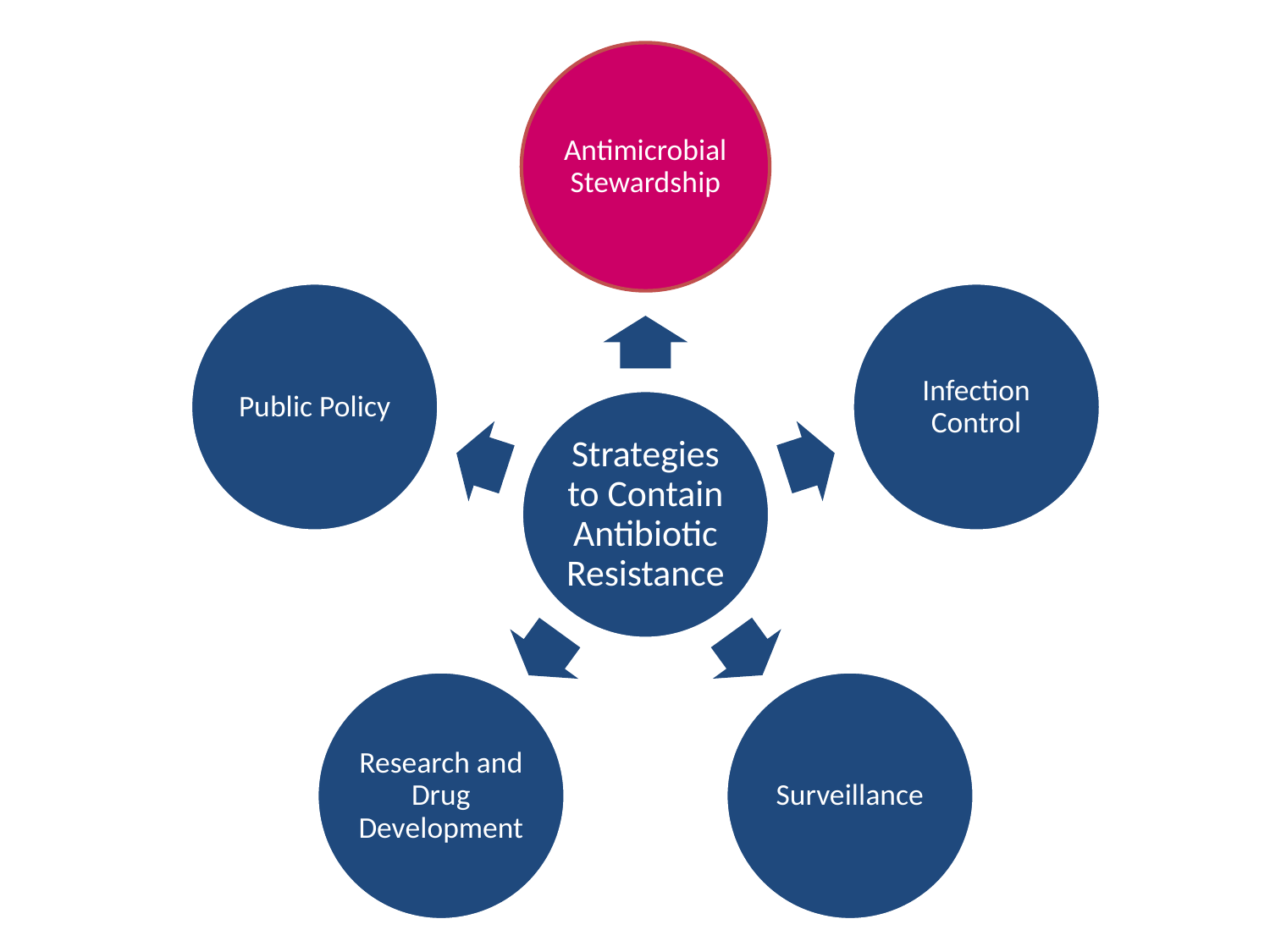

## Slide 13
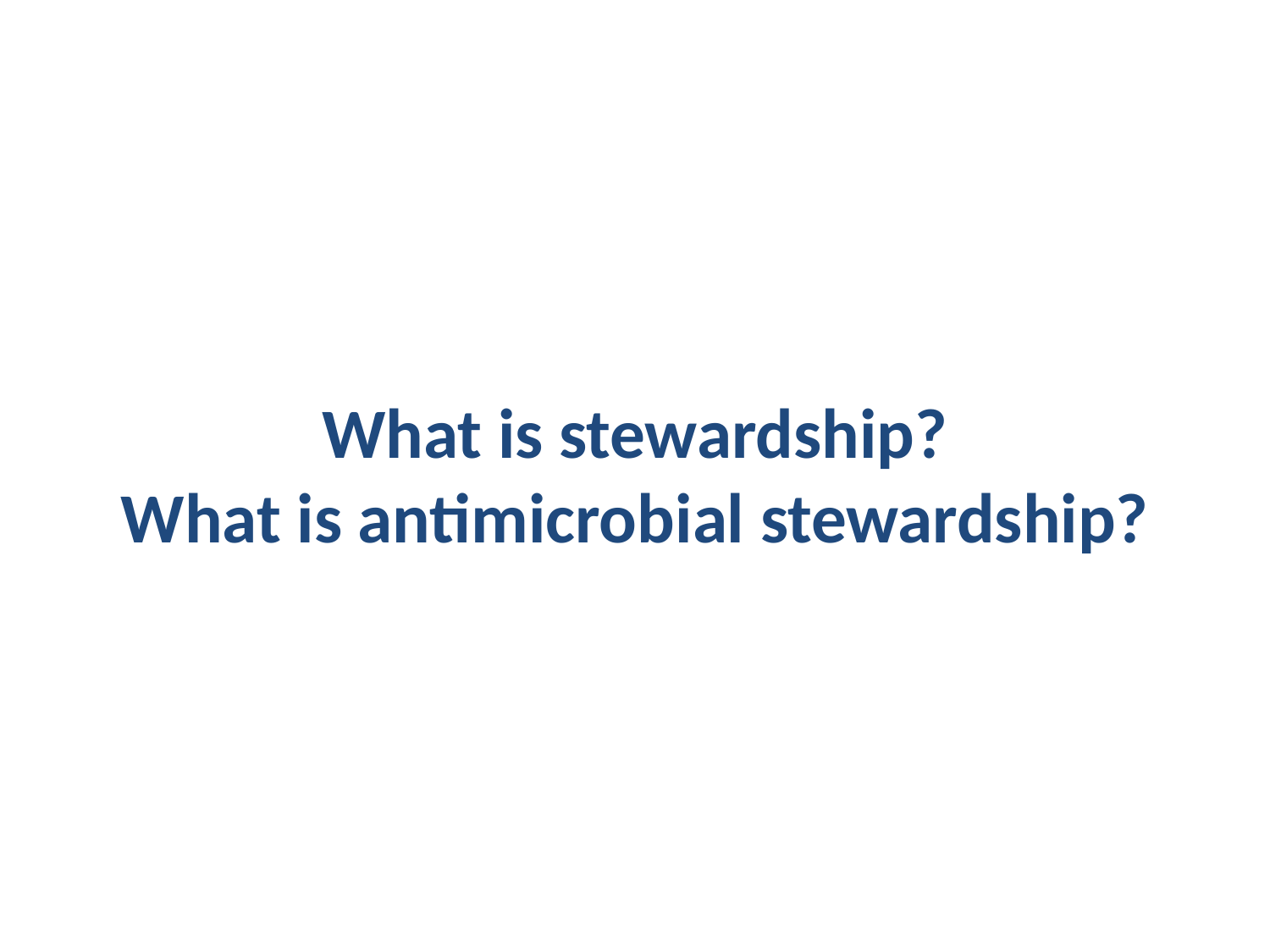

# What is stewardship? What is antimicrobial stewardship?

## Slide 14
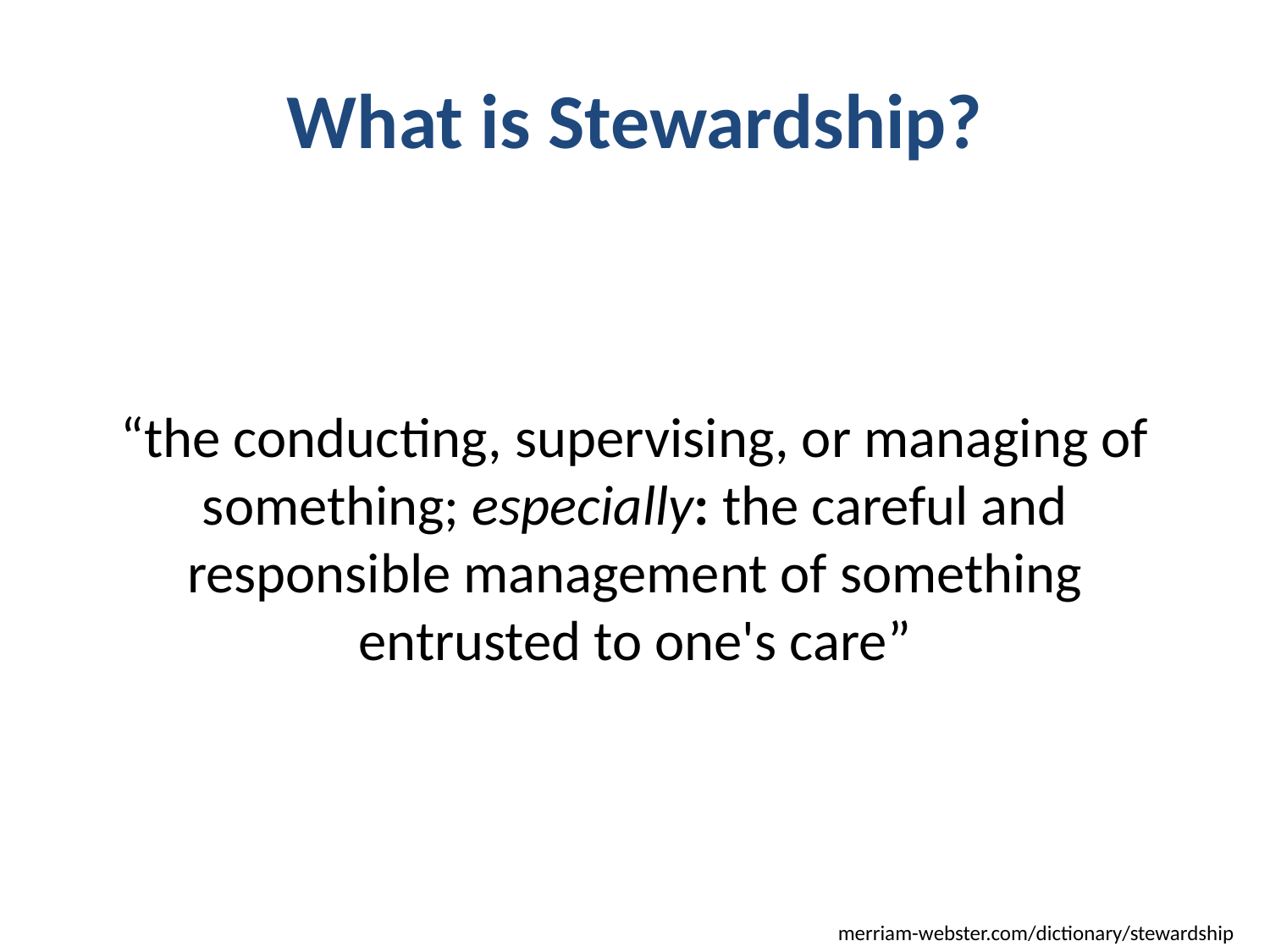

# What is Stewardship?
“the conducting, supervising, or managing of something; especially: the careful and responsible management of something entrusted to one's care”
merriam-webster.com/dictionary/stewardship

## Slide 15
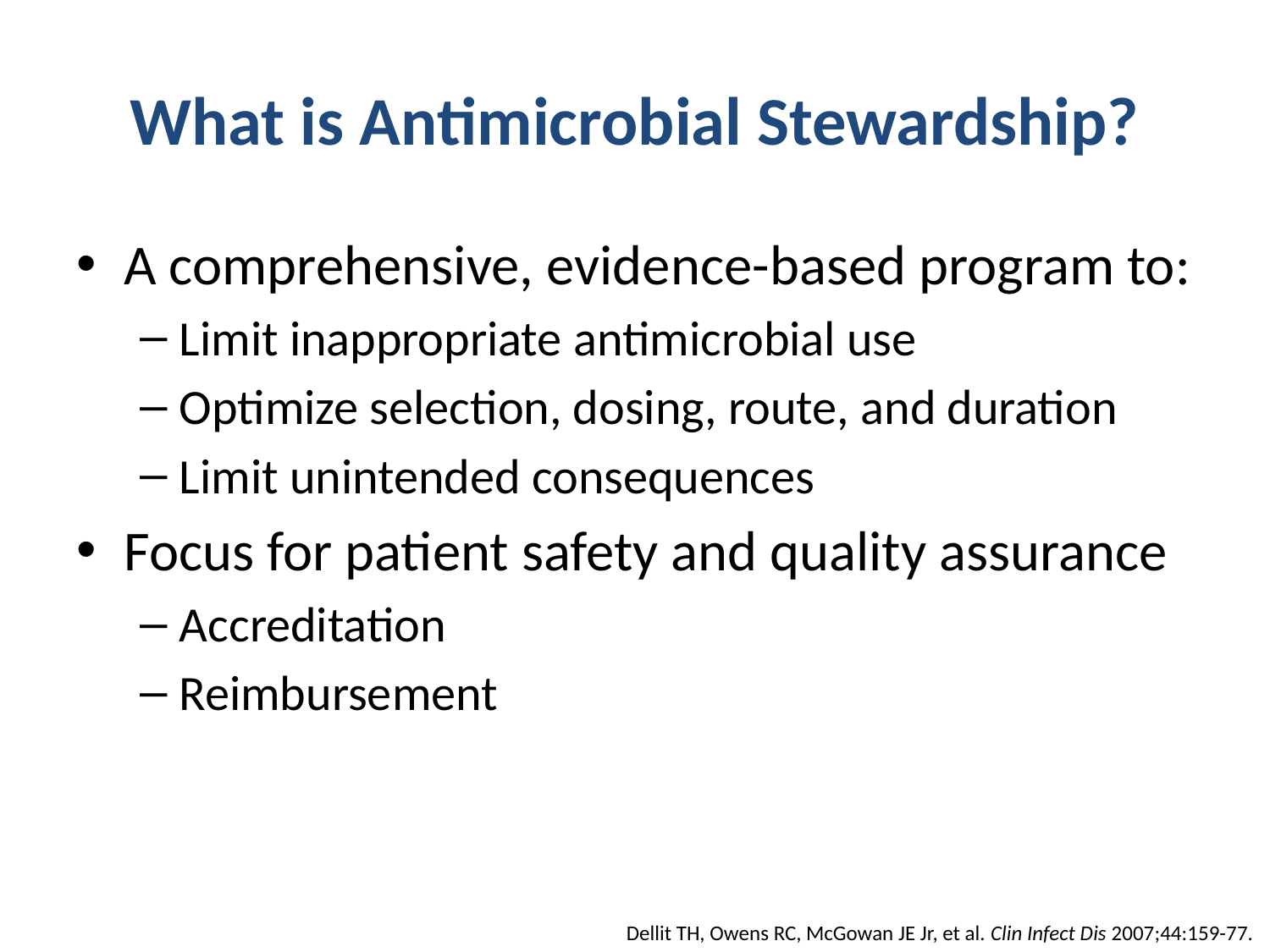

# What is Antimicrobial Stewardship?
A comprehensive, evidence-based program to:
Limit inappropriate antimicrobial use
Optimize selection, dosing, route, and duration
Limit unintended consequences
Focus for patient safety and quality assurance
Accreditation
Reimbursement
Dellit TH, Owens RC, McGowan JE Jr, et al. Clin Infect Dis 2007;44:159-77.

## Slide 16
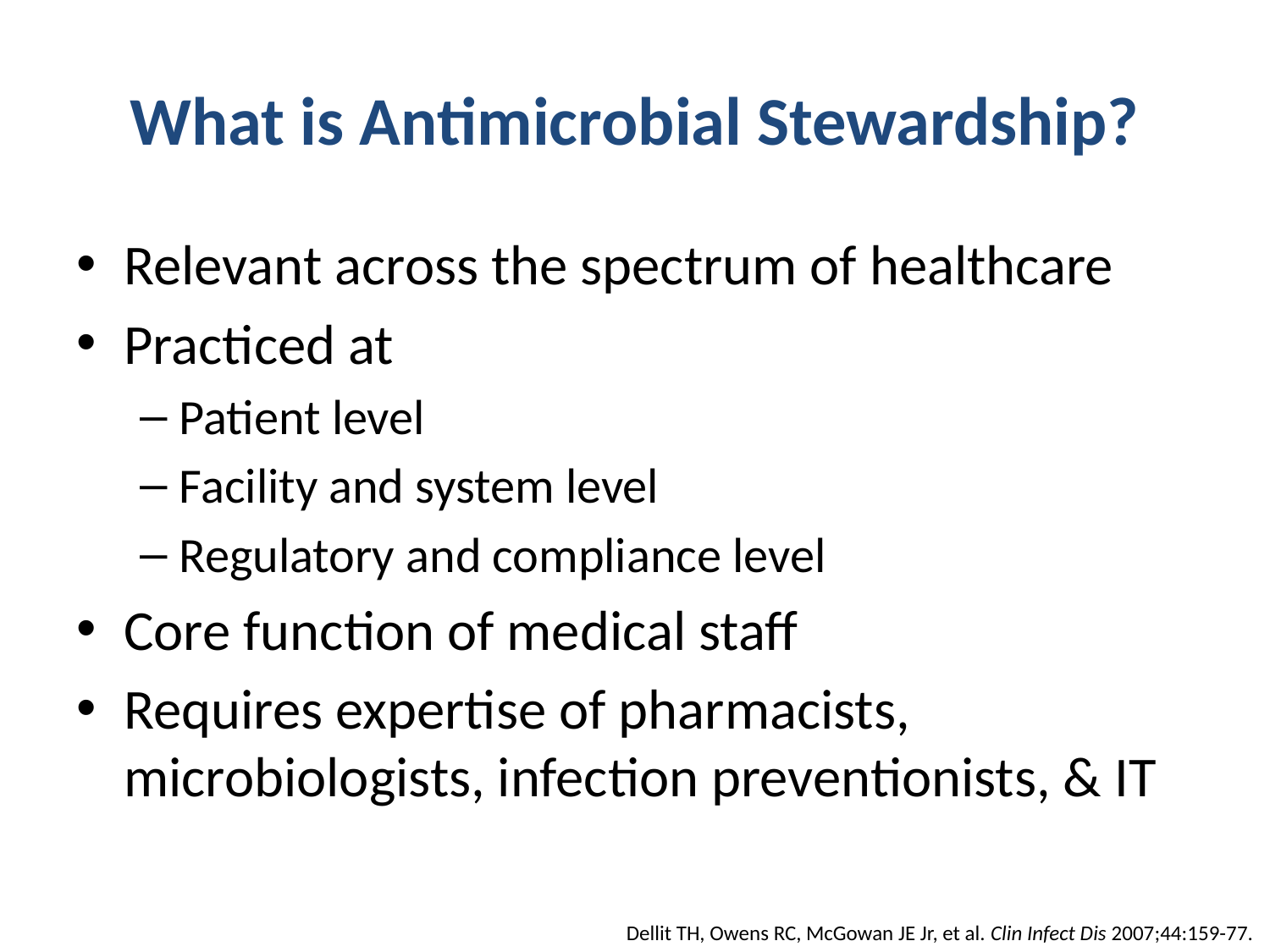

# What is Antimicrobial Stewardship?
Relevant across the spectrum of healthcare
Practiced at
Patient level
Facility and system level
Regulatory and compliance level
Core function of medical staff
Requires expertise of pharmacists, microbiologists, infection preventionists, & IT
Dellit TH, Owens RC, McGowan JE Jr, et al. Clin Infect Dis 2007;44:159-77.

## Slide 17
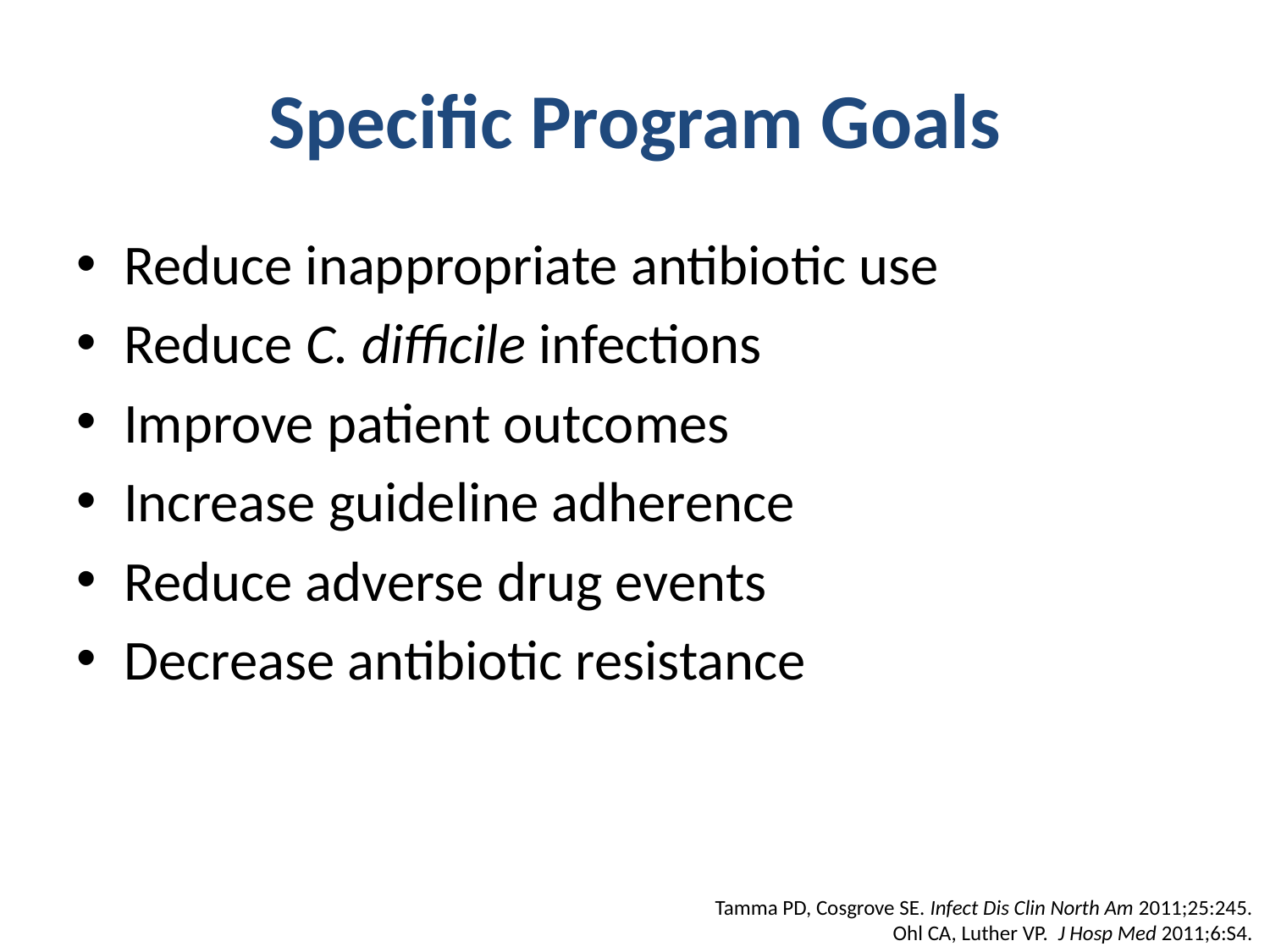

# Specific Program Goals
Reduce inappropriate antibiotic use
Reduce C. difficile infections
Improve patient outcomes
Increase guideline adherence
Reduce adverse drug events
Decrease antibiotic resistance
Tamma PD, Cosgrove SE. Infect Dis Clin North Am 2011;25:245.
Ohl CA, Luther VP. J Hosp Med 2011;6:S4.

## Slide 18
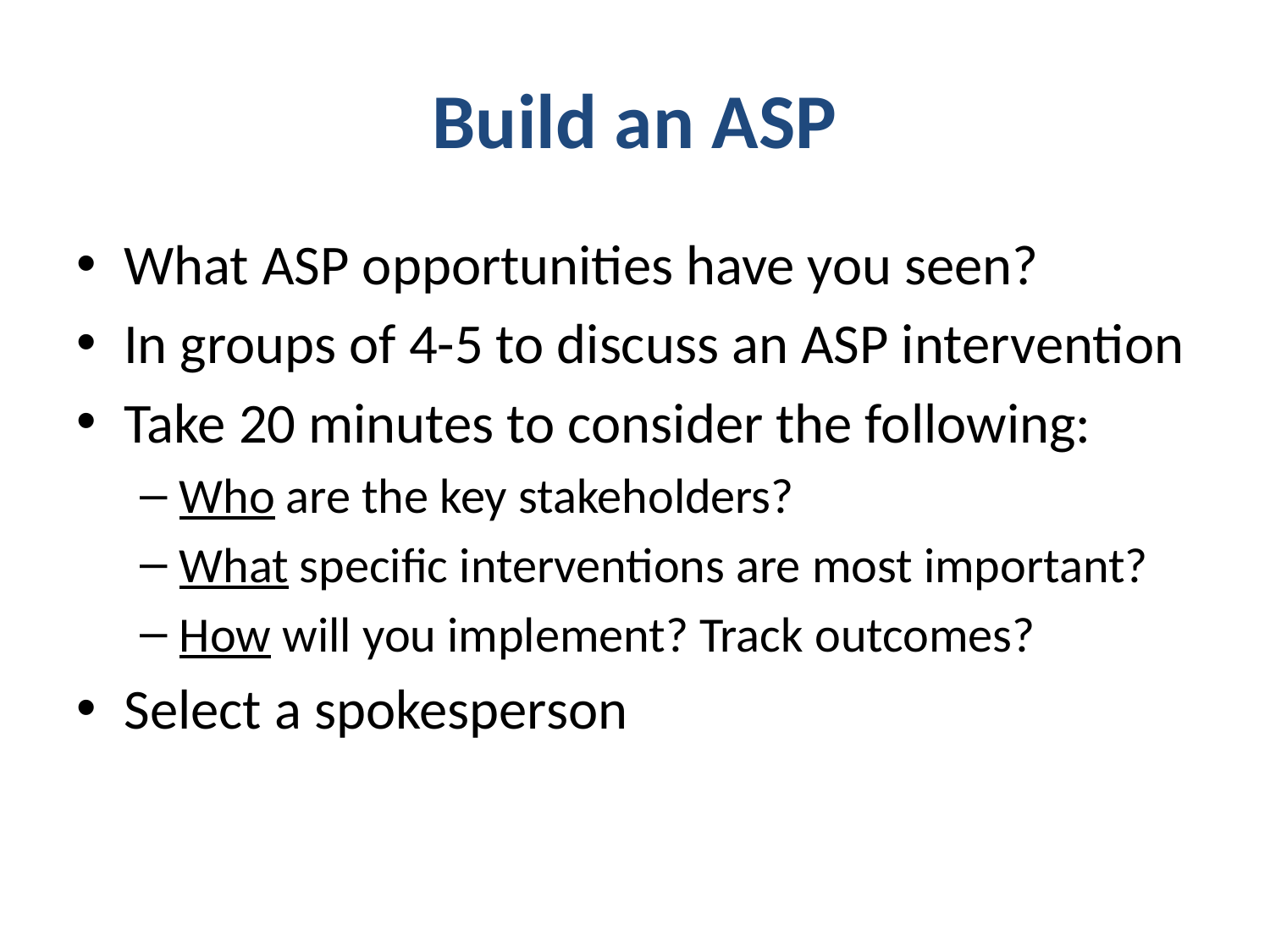

# Build an ASP
What ASP opportunities have you seen?
In groups of 4-5 to discuss an ASP intervention
Take 20 minutes to consider the following:
Who are the key stakeholders?
What specific interventions are most important?
How will you implement? Track outcomes?
Select a spokesperson

## Slide 19
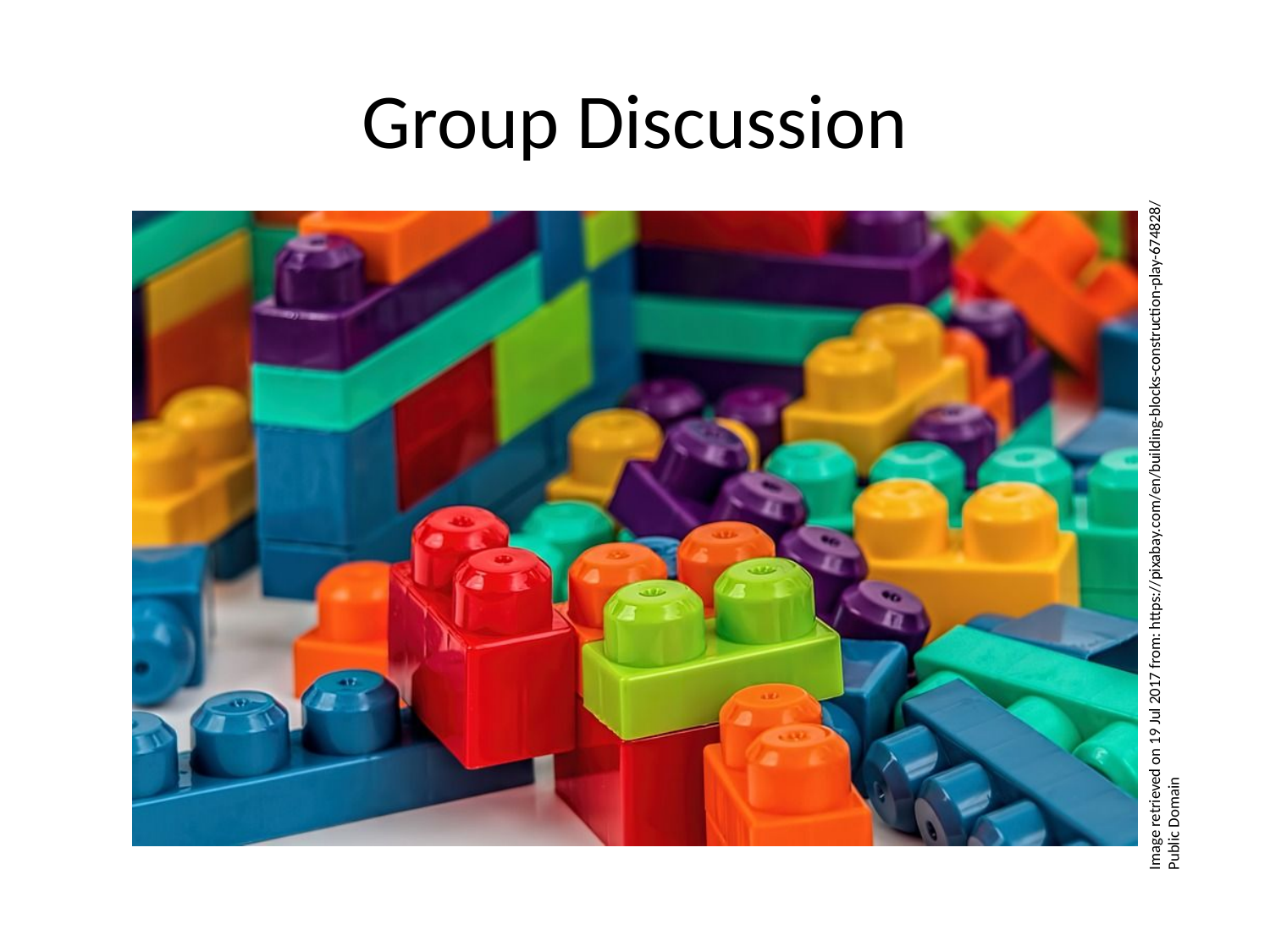

# Group Discussion
Image retrieved on 19 Jul 2017 from: https://pixabay.com/en/building-blocks-construction-play-674828/
Public Domain

## Slide 20
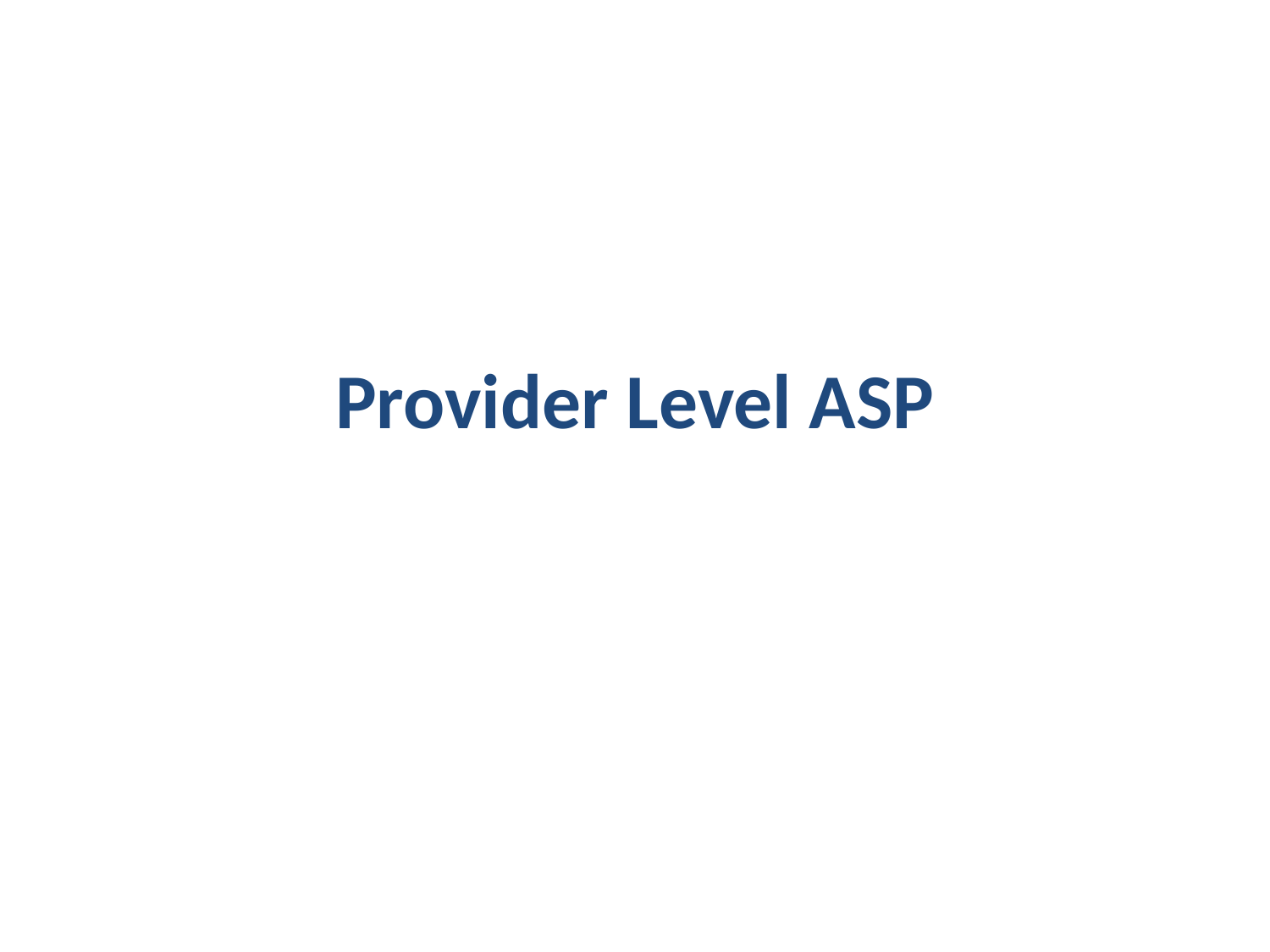

# Provider Level ASP

## Slide 21
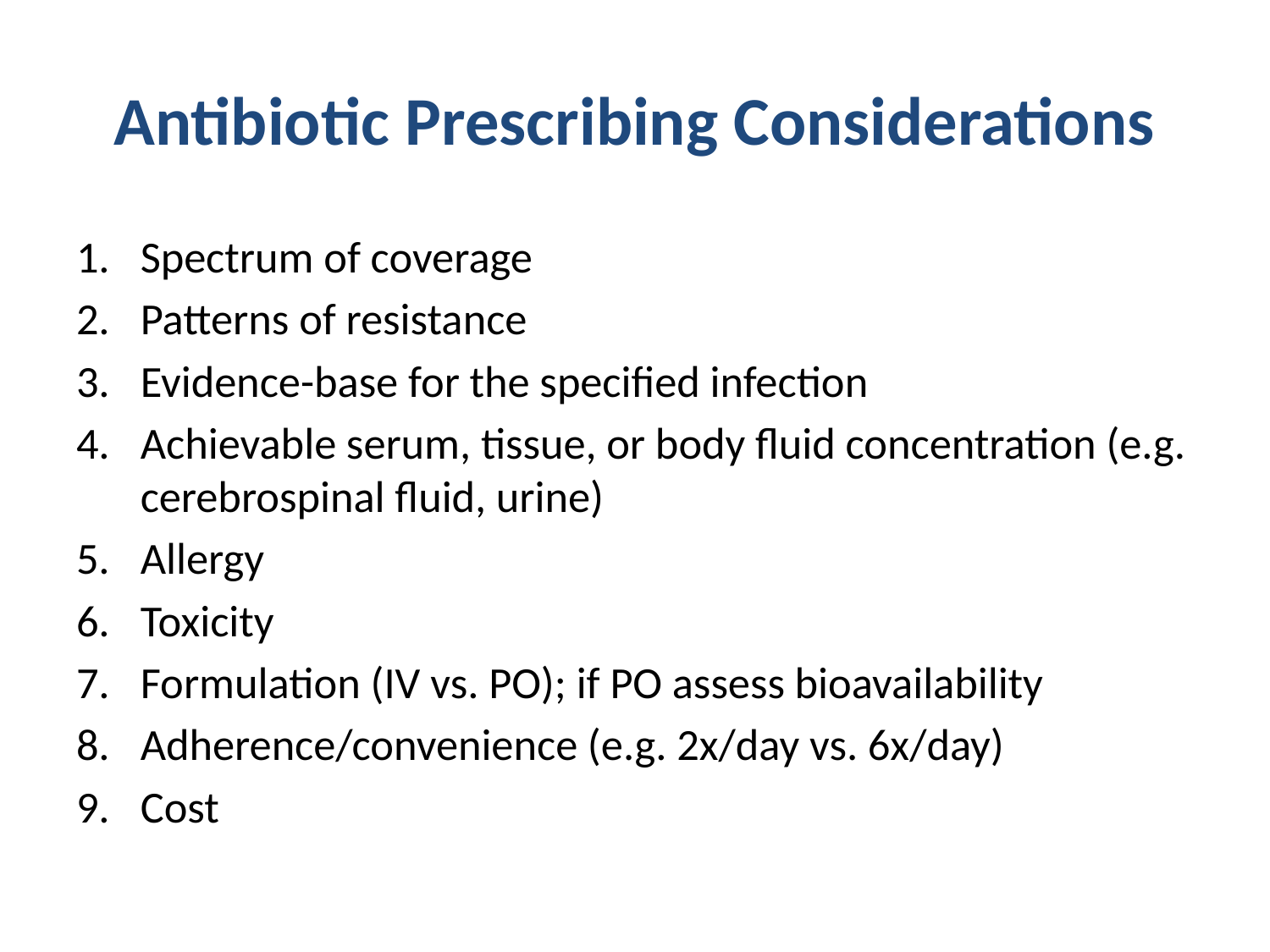

# Antibiotic Prescribing Considerations
Spectrum of coverage
Patterns of resistance
Evidence-base for the specified infection
Achievable serum, tissue, or body fluid concentration (e.g. cerebrospinal fluid, urine)
Allergy
Toxicity
Formulation (IV vs. PO); if PO assess bioavailability
Adherence/convenience (e.g. 2x/day vs. 6x/day)
Cost

## Slide 22
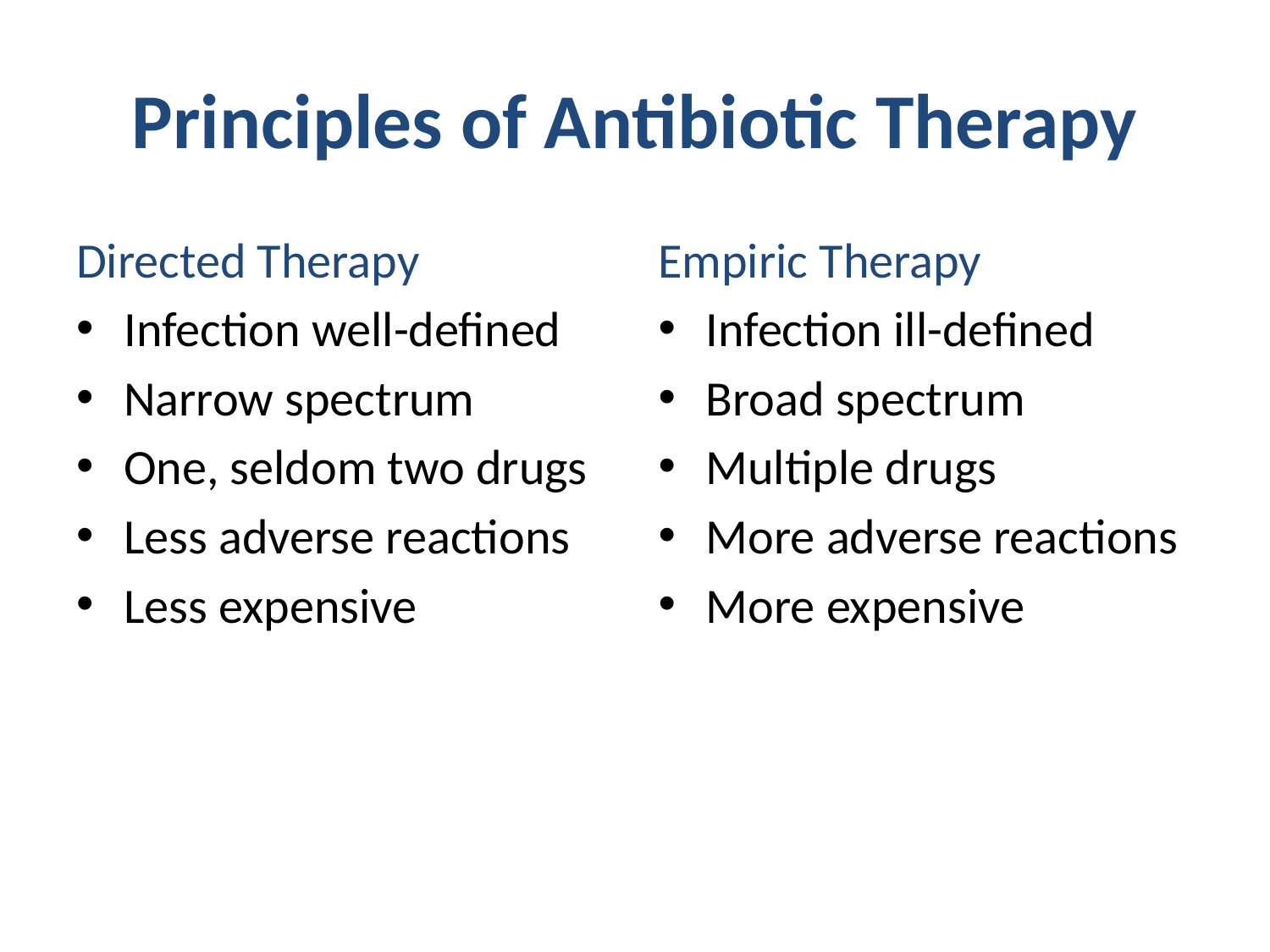

# Principles of Antibiotic Therapy
Directed Therapy
Infection well-defined
Narrow spectrum
One, seldom two drugs
Less adverse reactions
Less expensive
Empiric Therapy
Infection ill-defined
Broad spectrum
Multiple drugs
More adverse reactions
More expensive

## Slide 23
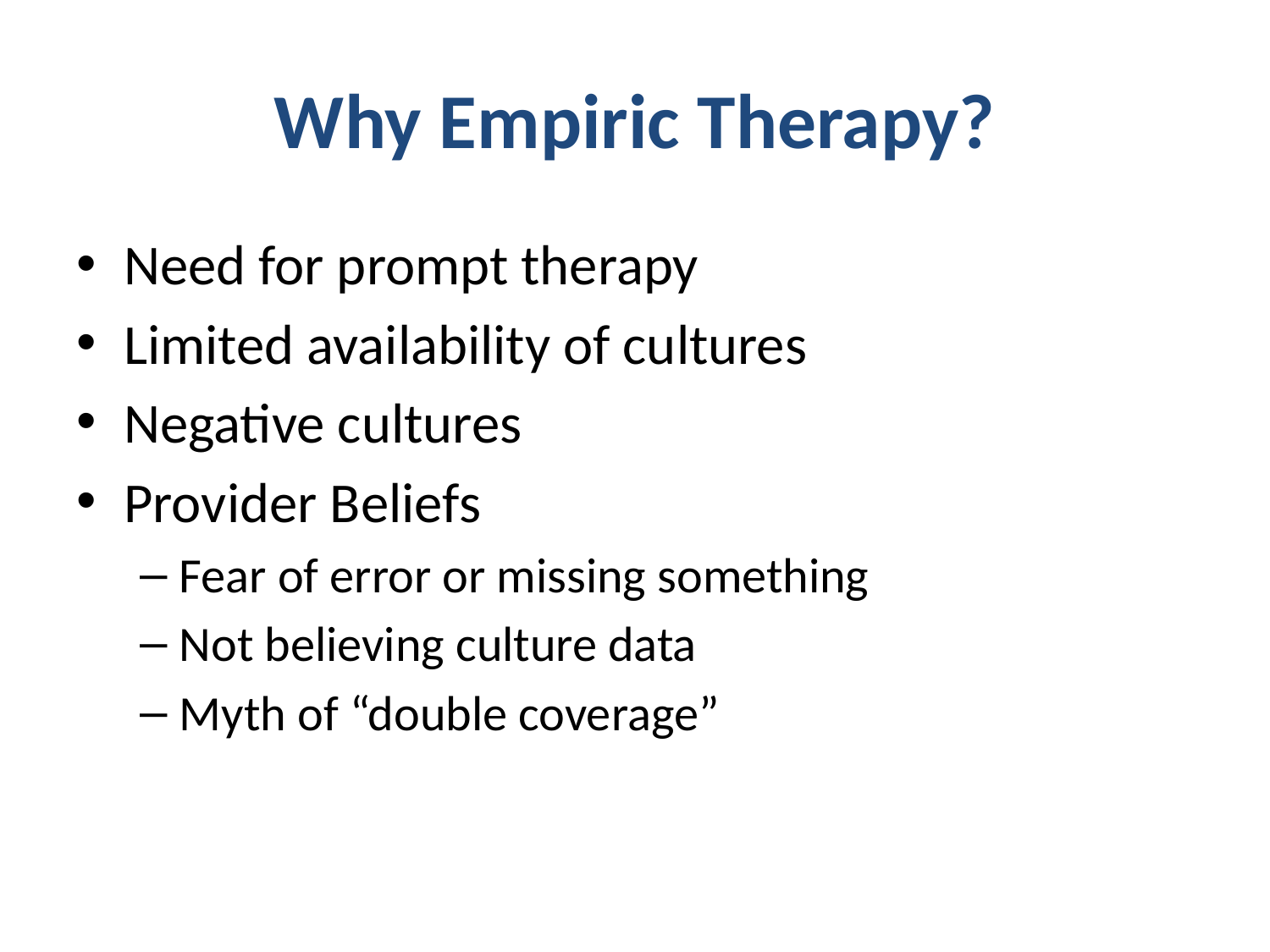

# Why Empiric Therapy?
Need for prompt therapy
Limited availability of cultures
Negative cultures
Provider Beliefs
Fear of error or missing something
Not believing culture data
Myth of “double coverage”

## Slide 24
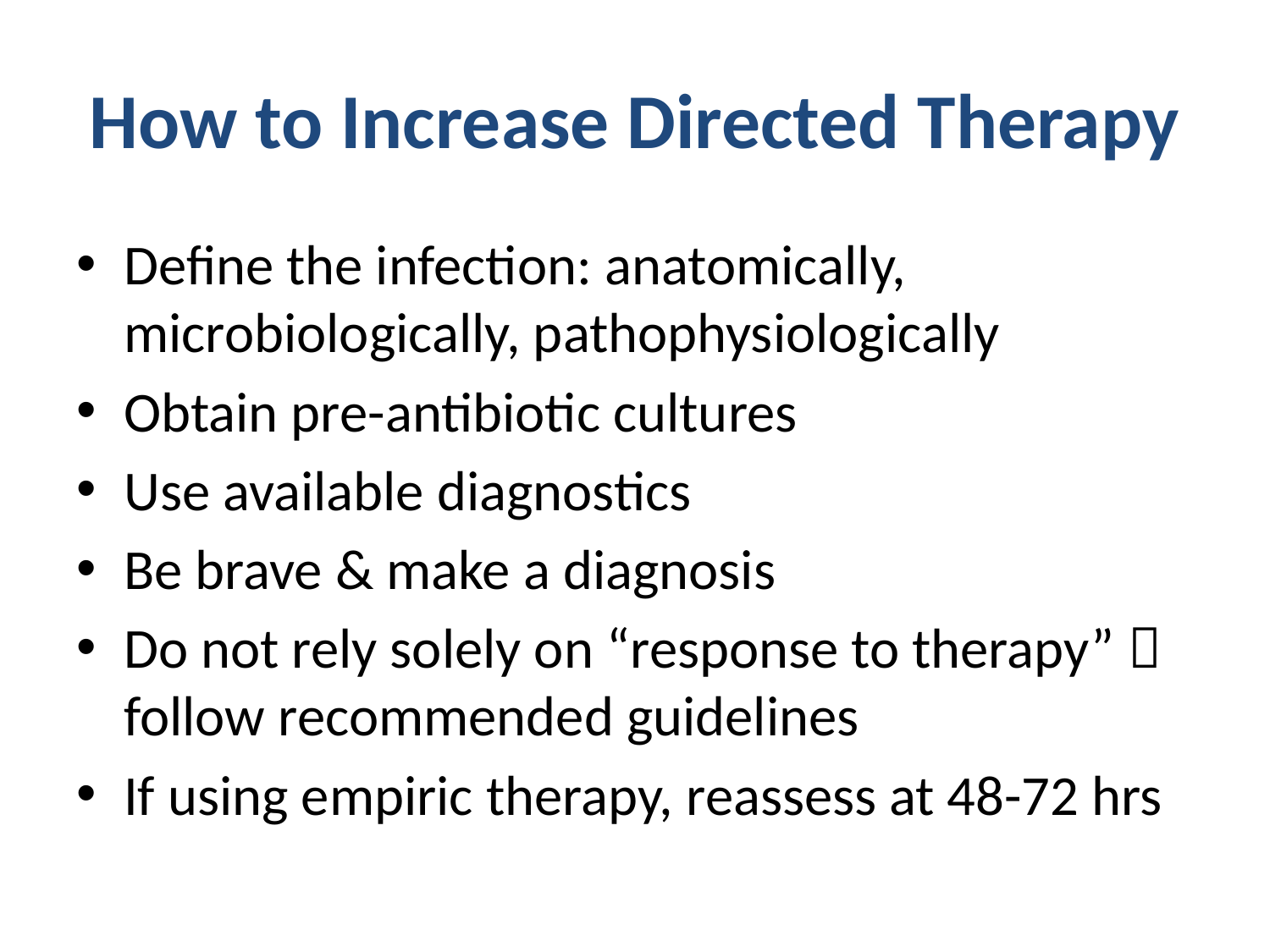

# How to Increase Directed Therapy
Define the infection: anatomically, microbiologically, pathophysiologically
Obtain pre-antibiotic cultures
Use available diagnostics
Be brave & make a diagnosis
Do not rely solely on “response to therapy”  follow recommended guidelines
If using empiric therapy, reassess at 48-72 hrs

## Slide 25
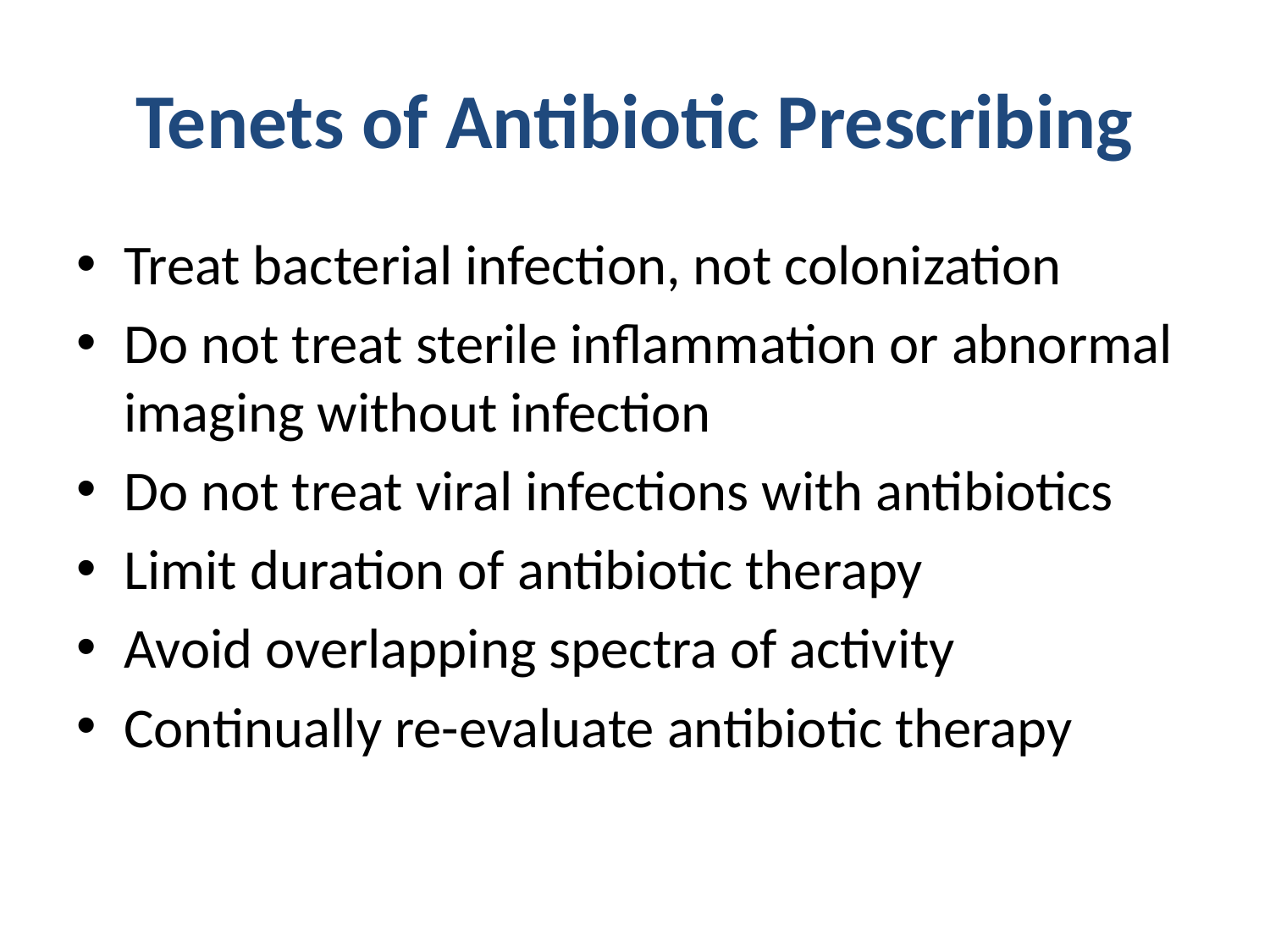

# Tenets of Antibiotic Prescribing
Treat bacterial infection, not colonization
Do not treat sterile inflammation or abnormal imaging without infection
Do not treat viral infections with antibiotics
Limit duration of antibiotic therapy
Avoid overlapping spectra of activity
Continually re-evaluate antibiotic therapy

## Slide 26
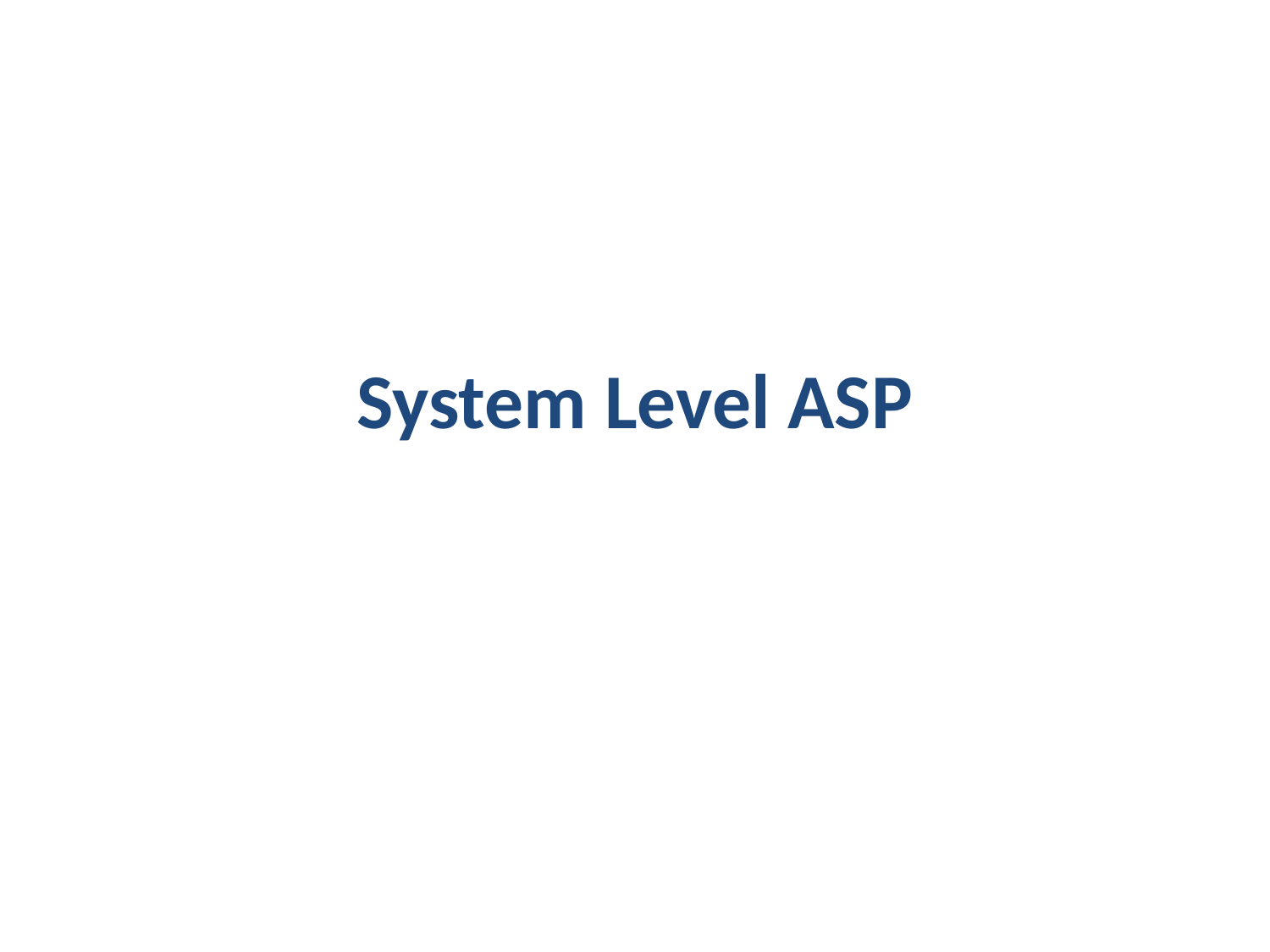

# System Level ASP

## Slide 27
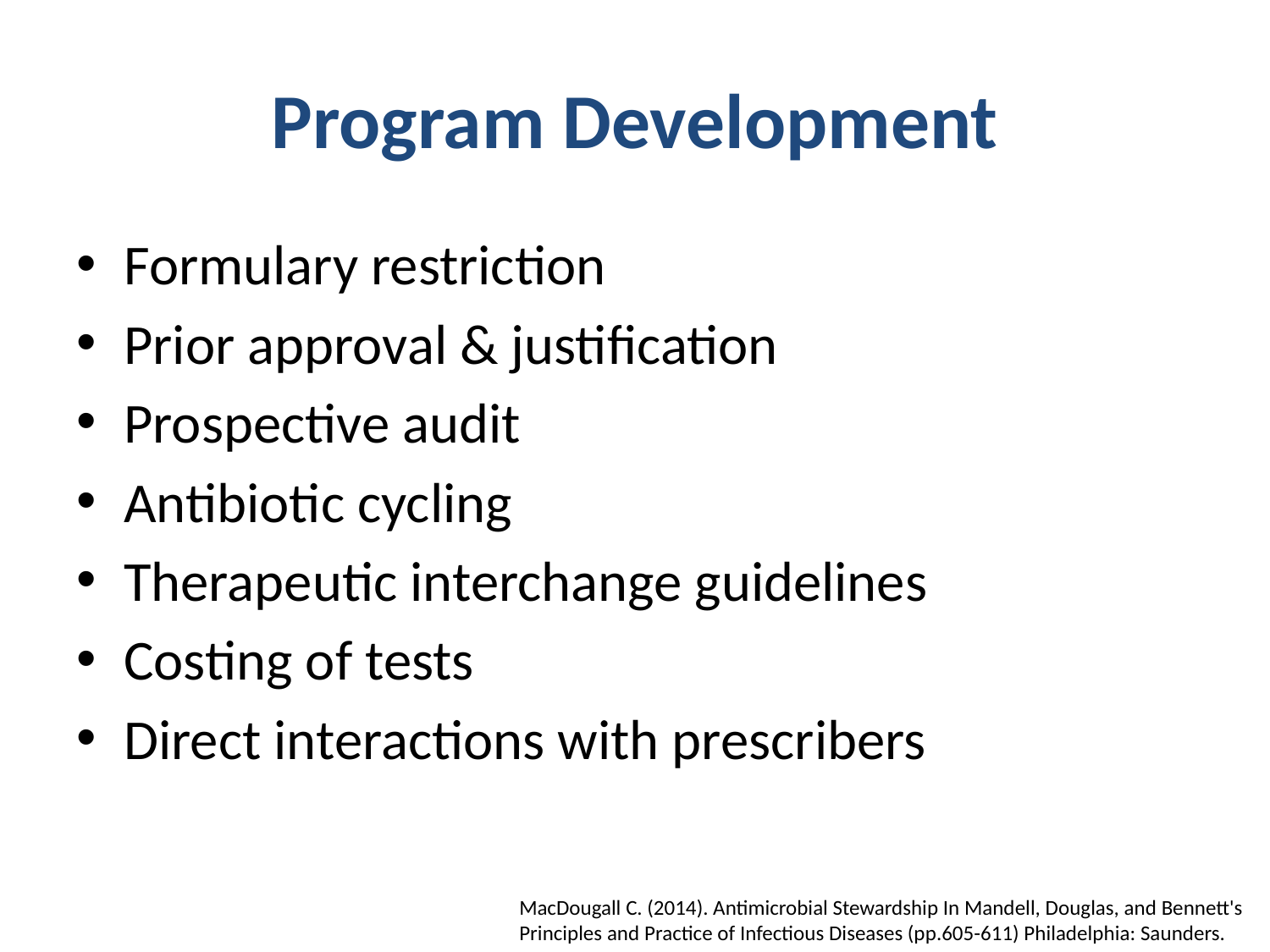

# Program Development
Formulary restriction
Prior approval & justification
Prospective audit
Antibiotic cycling
Therapeutic interchange guidelines
Costing of tests
Direct interactions with prescribers
MacDougall C. (2014). Antimicrobial Stewardship In Mandell, Douglas, and Bennett's Principles and Practice of Infectious Diseases (pp.605-611) Philadelphia: Saunders.

## Slide 28
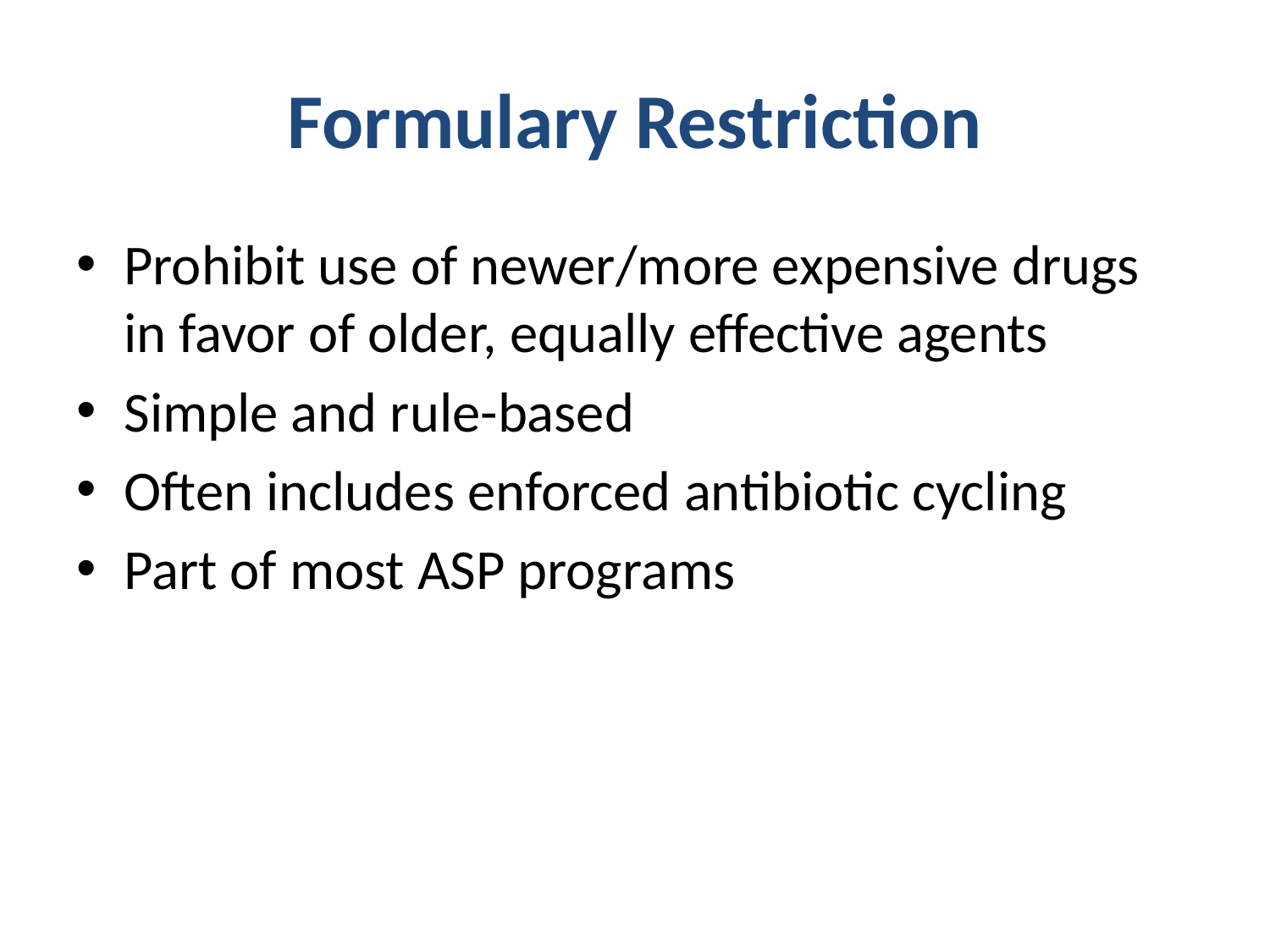

# Formulary Restriction
Prohibit use of newer/more expensive drugs in favor of older, equally effective agents
Simple and rule-based
Often includes enforced antibiotic cycling
Part of most ASP programs

## Slide 29
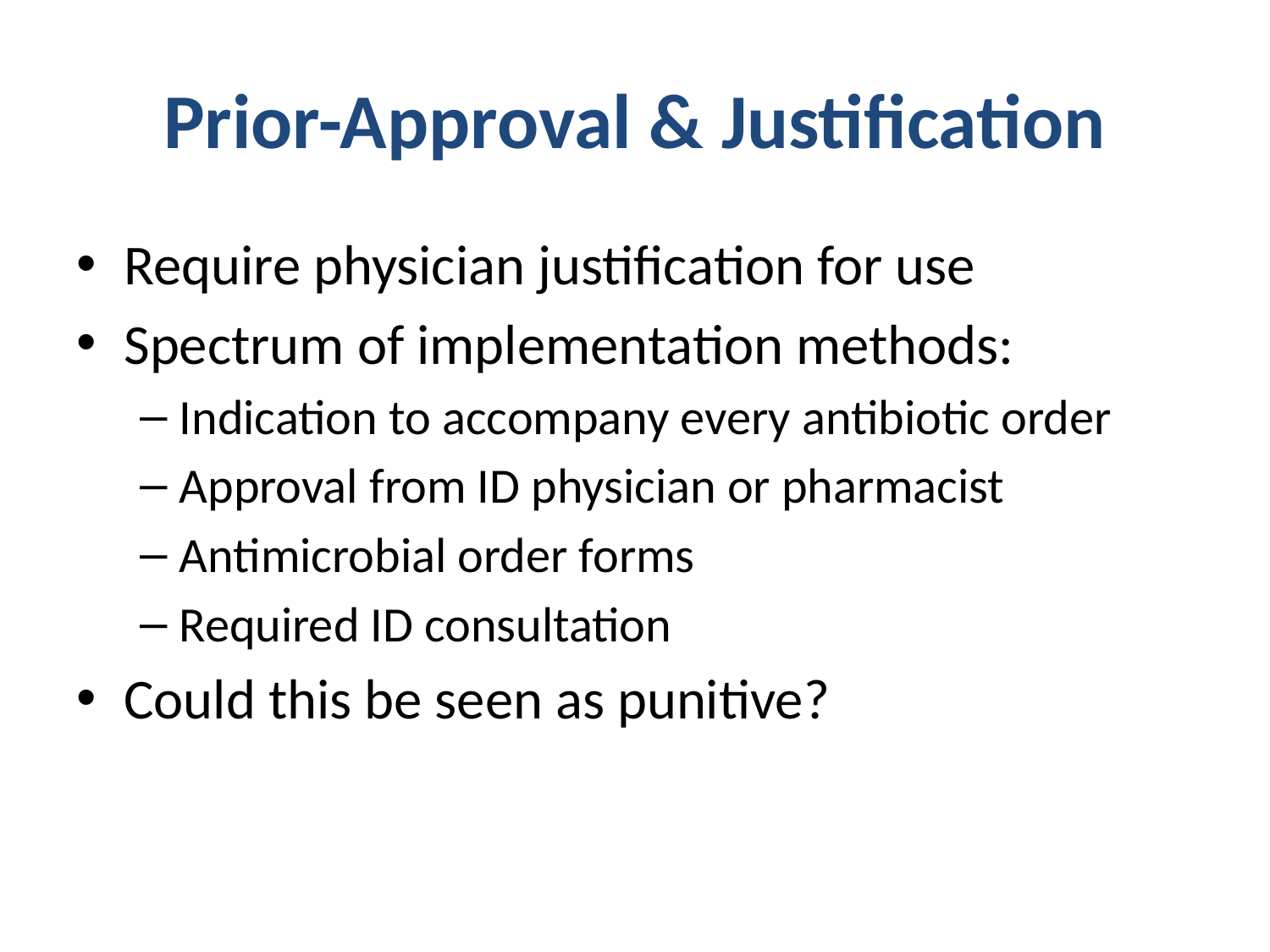

# Prior-Approval & Justification
Require physician justification for use
Spectrum of implementation methods:
Indication to accompany every antibiotic order
Approval from ID physician or pharmacist
Antimicrobial order forms
Required ID consultation
Could this be seen as punitive?

## Slide 30
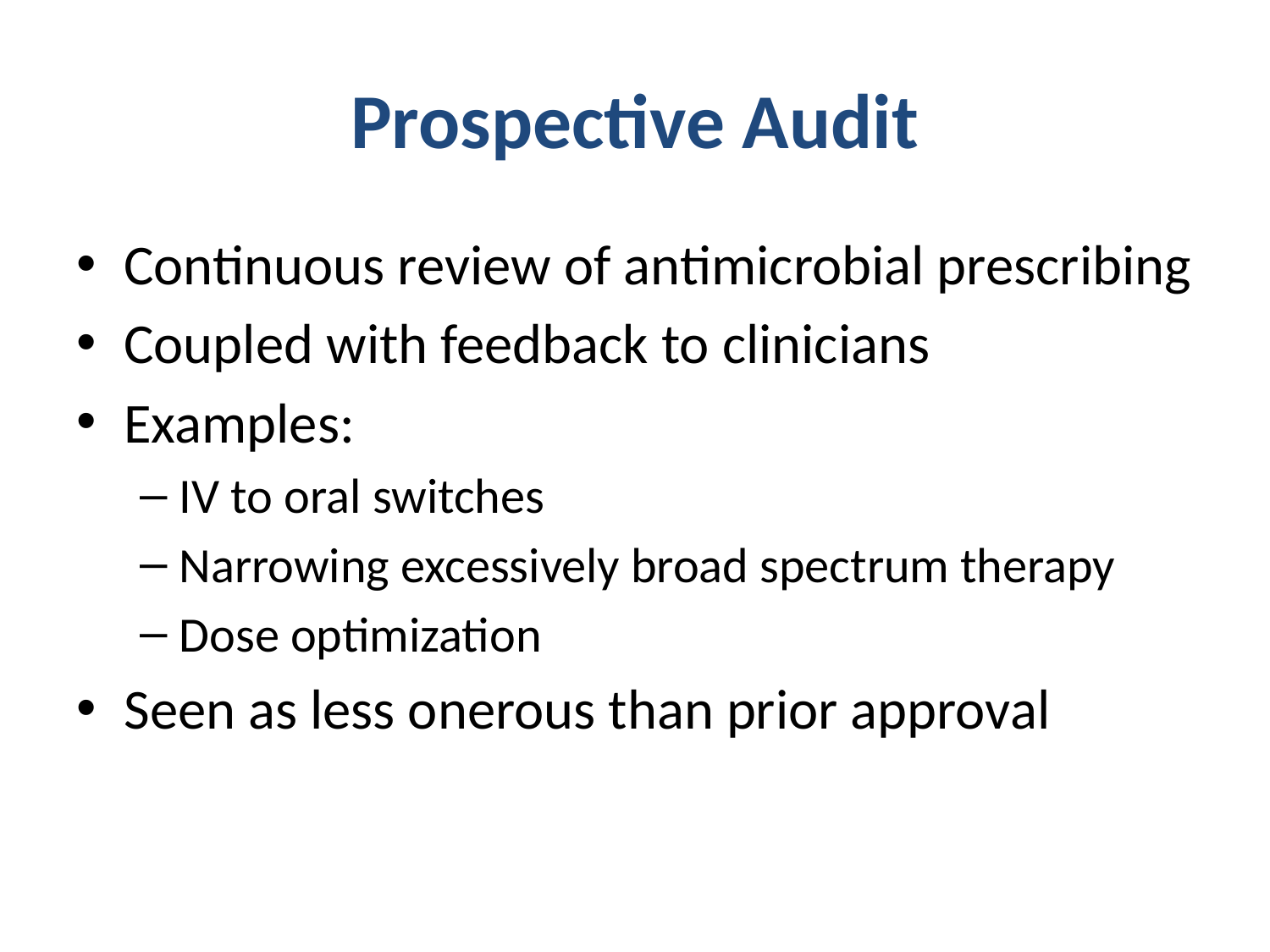

# Prospective Audit
Continuous review of antimicrobial prescribing
Coupled with feedback to clinicians
Examples:
IV to oral switches
Narrowing excessively broad spectrum therapy
Dose optimization
Seen as less onerous than prior approval

## Slide 31
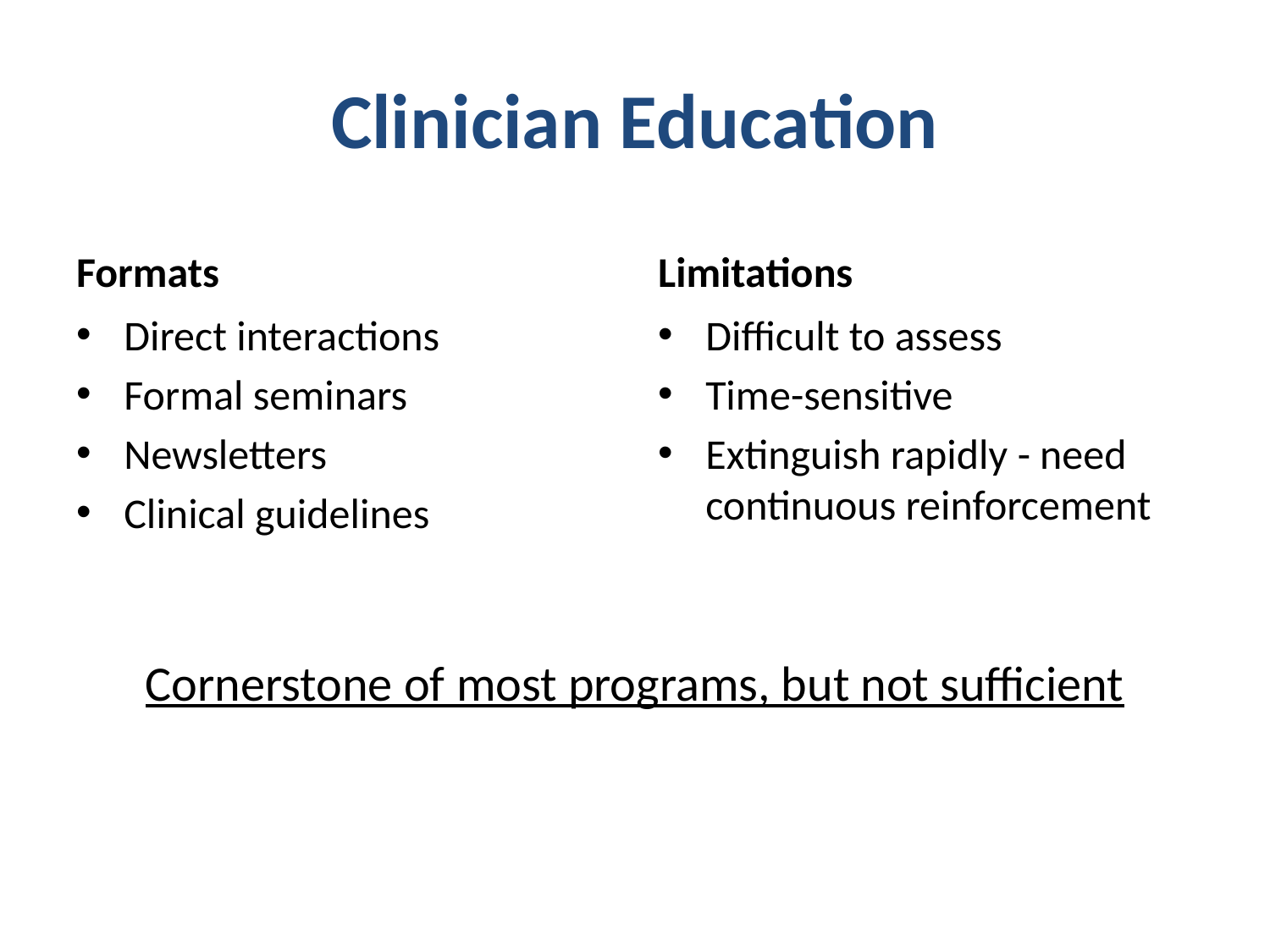

# Clinician Education
Formats
Limitations
Direct interactions
Formal seminars
Newsletters
Clinical guidelines
Difficult to assess
Time-sensitive
Extinguish rapidly - need continuous reinforcement
Cornerstone of most programs, but not sufficient

## Slide 32
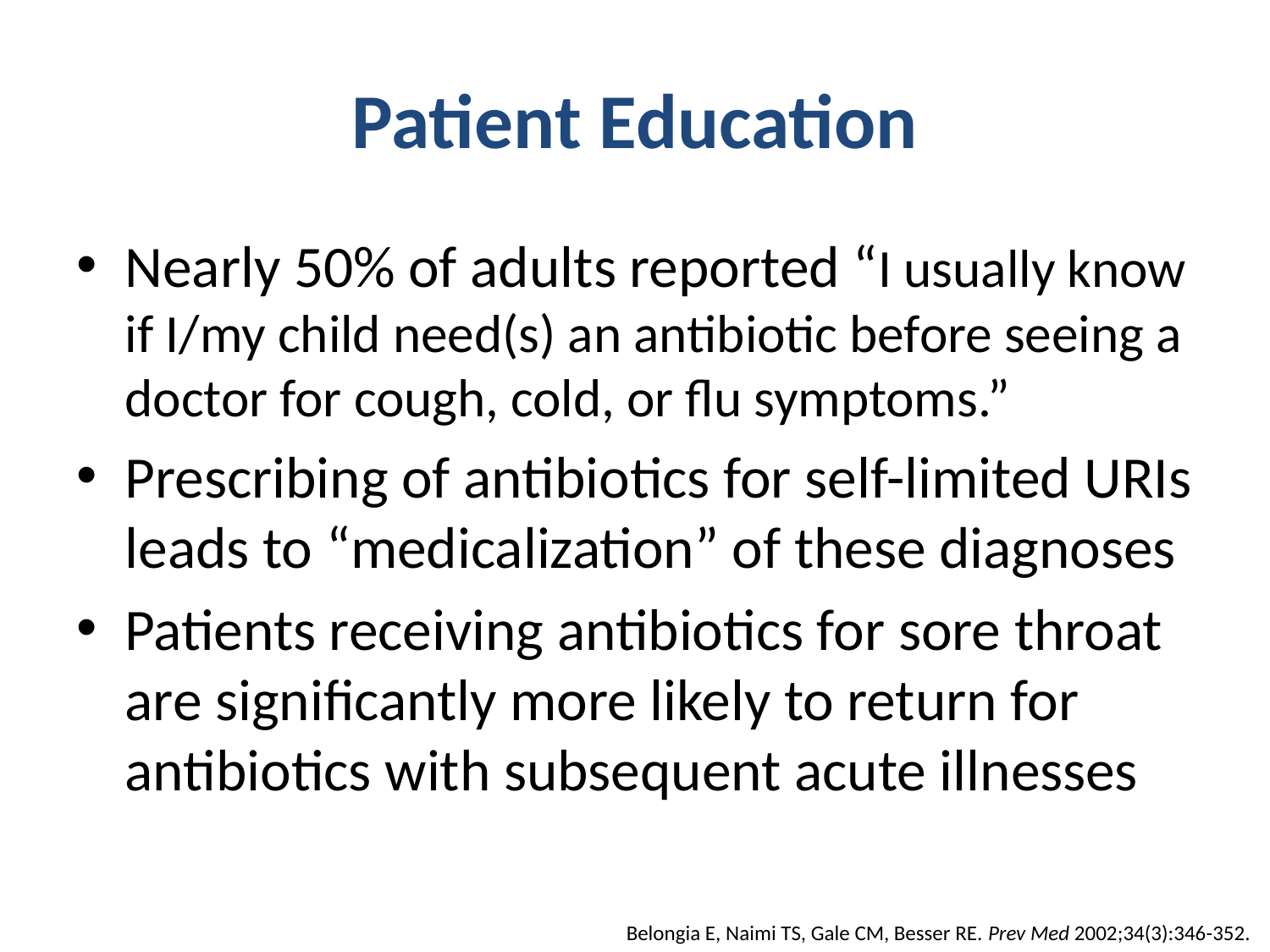

# Patient Education
Nearly 50% of adults reported “I usually know if I/my child need(s) an antibiotic before seeing a doctor for cough, cold, or flu symptoms.”
Prescribing of antibiotics for self-limited URIs leads to “medicalization” of these diagnoses
Patients receiving antibiotics for sore throat are significantly more likely to return for antibiotics with subsequent acute illnesses
Belongia E, Naimi TS, Gale CM, Besser RE. Prev Med 2002;34(3):346-352.

## Slide 33
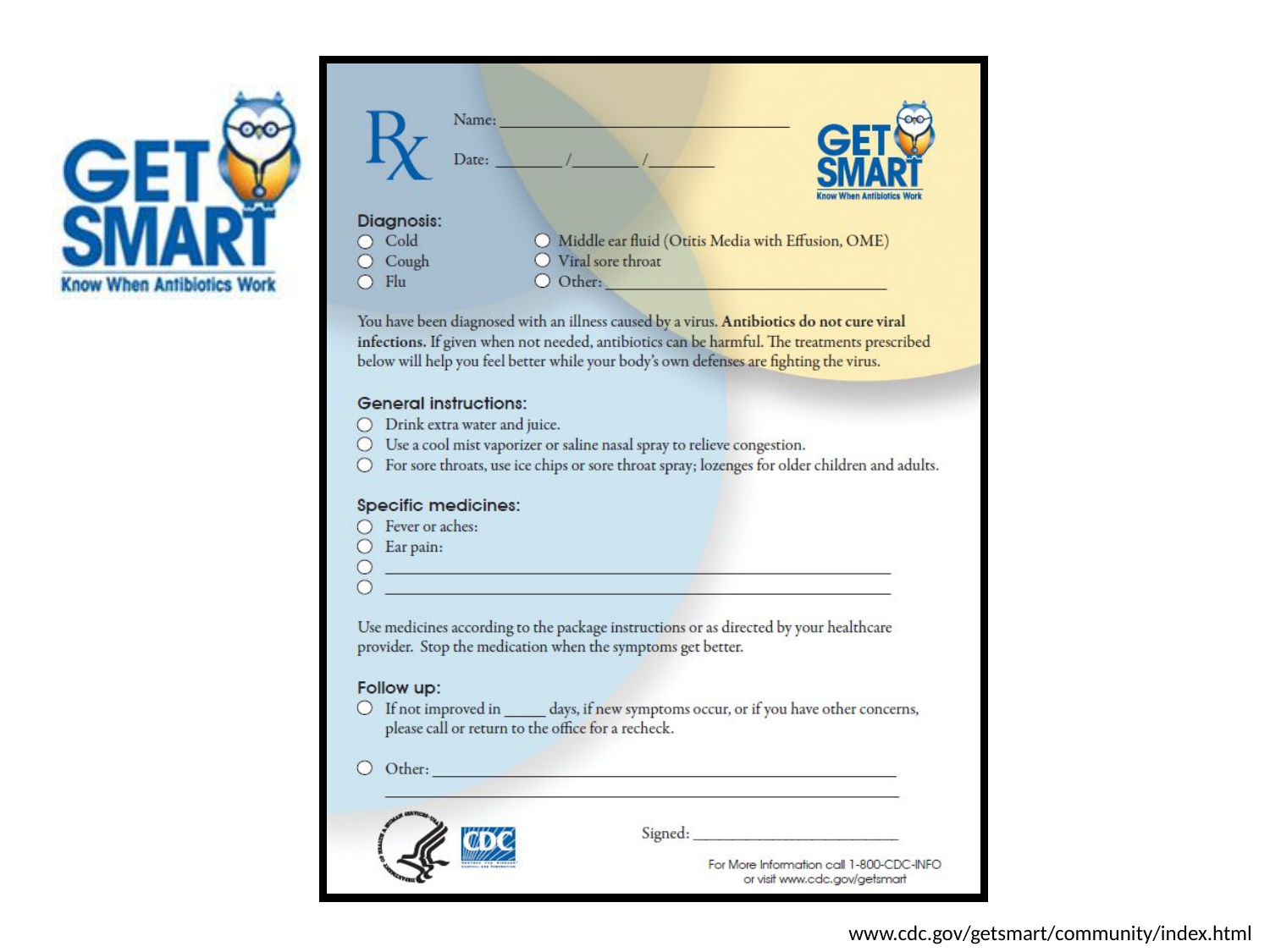

www.cdc.gov/getsmart/community/index.html

## Slide 34
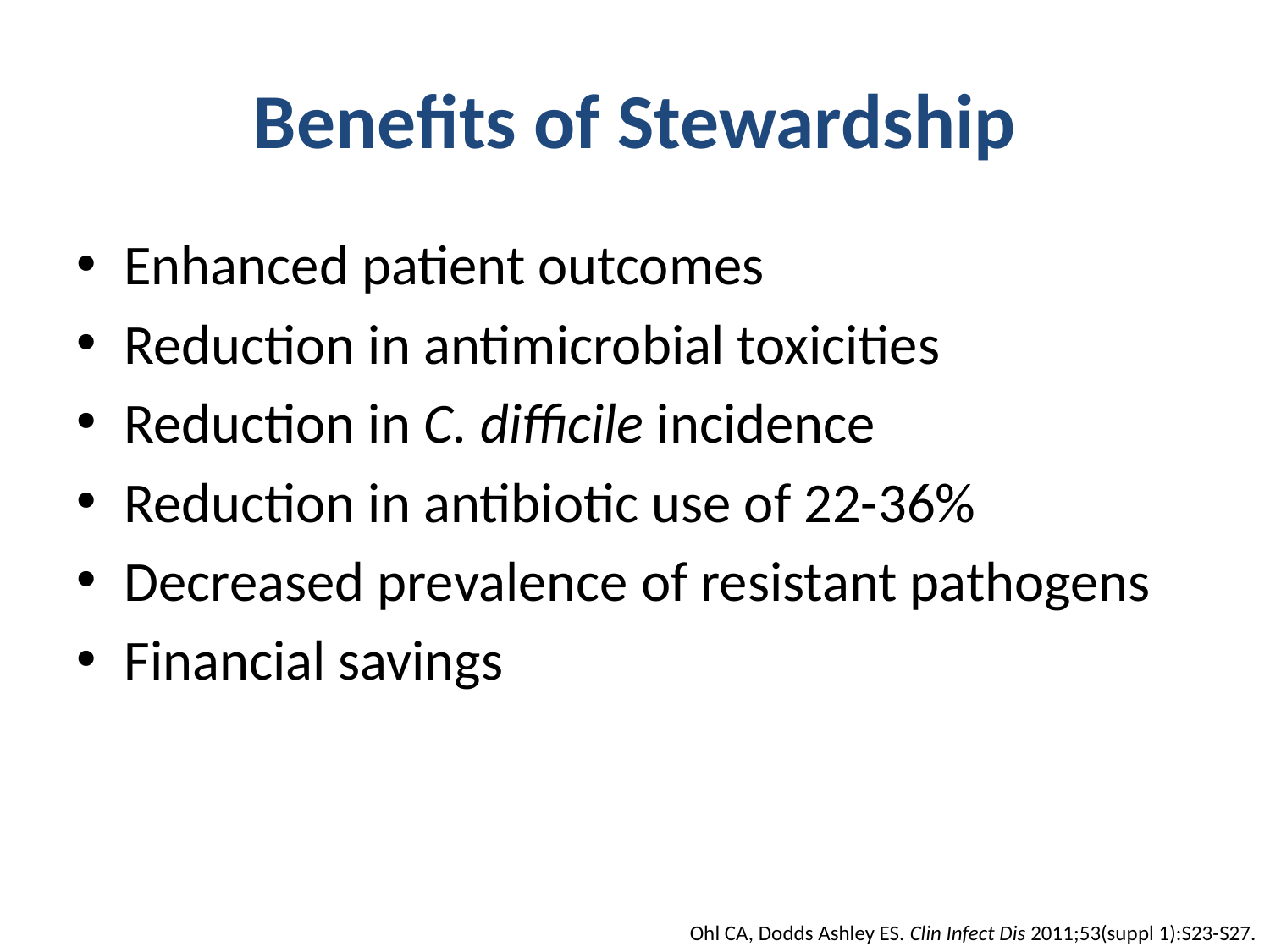

# Benefits of Stewardship
Enhanced patient outcomes
Reduction in antimicrobial toxicities
Reduction in C. difficile incidence
Reduction in antibiotic use of 22-36%
Decreased prevalence of resistant pathogens
Financial savings
Ohl CA, Dodds Ashley ES. Clin Infect Dis 2011;53(suppl 1):S23-S27.

## Slide 35
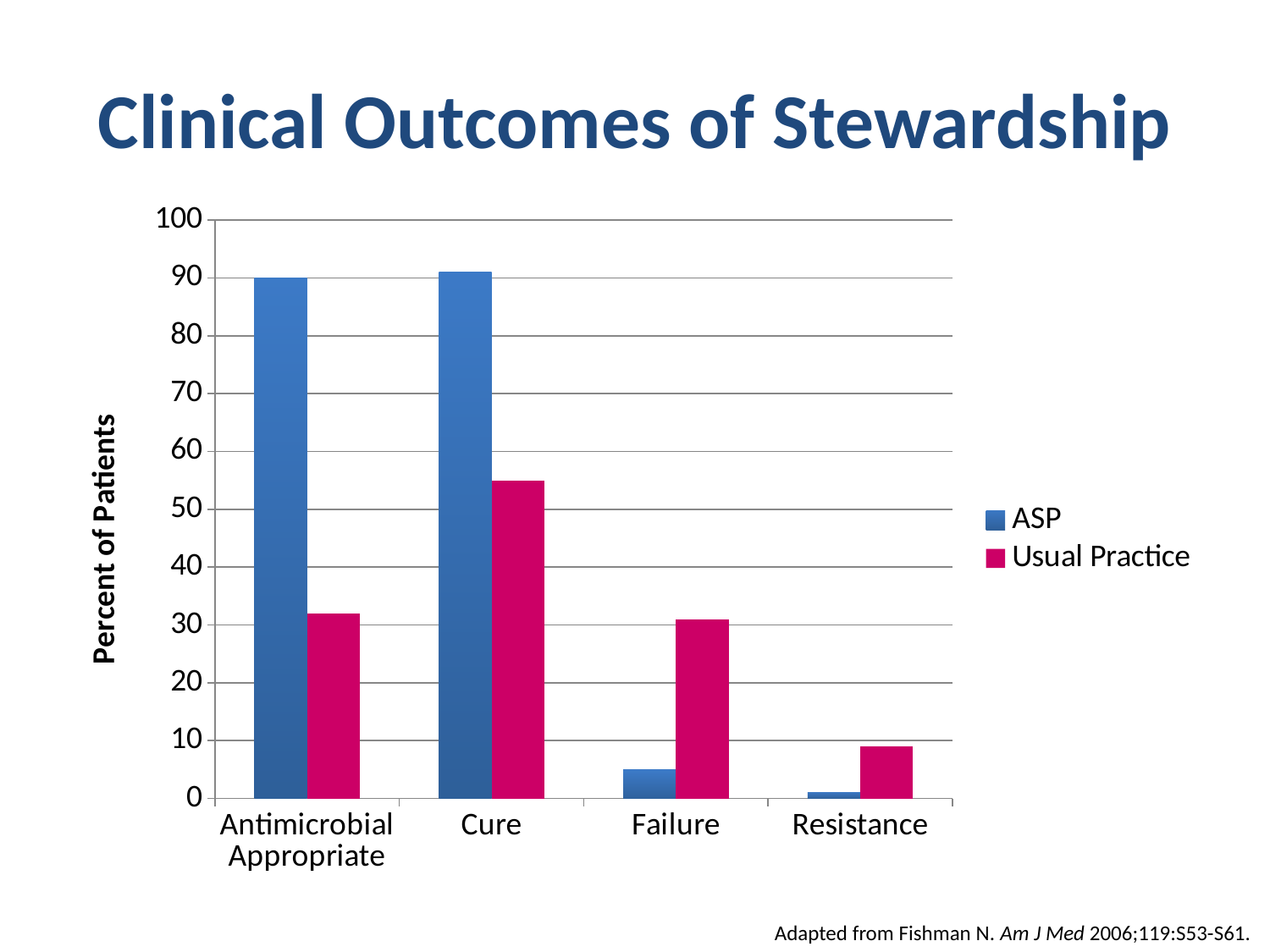

# Clinical Outcomes of Stewardship
### Chart
| Category | ASP | Usual Practice |
|---|---|---|
| Antimicrobial Appropriate | 90.0 | 32.0 |
| Cure | 91.0 | 55.0 |
| Failure | 5.0 | 31.0 |
| Resistance | 1.0 | 9.0 |Adapted from Fishman N. Am J Med 2006;119:S53-S61.

## Slide 36
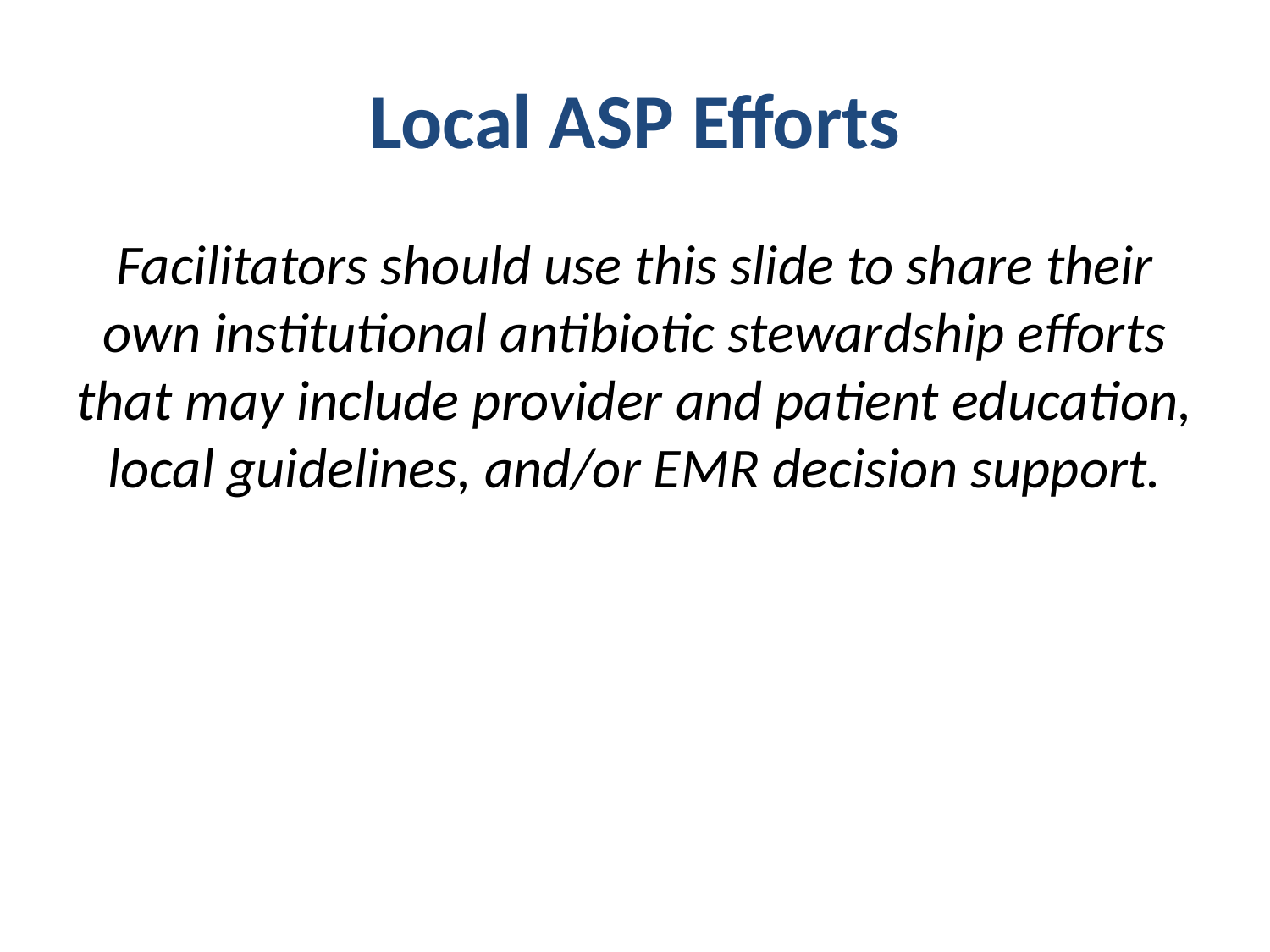

# Local ASP Efforts
Facilitators should use this slide to share their own institutional antibiotic stewardship efforts that may include provider and patient education, local guidelines, and/or EMR decision support.

## Slide 37
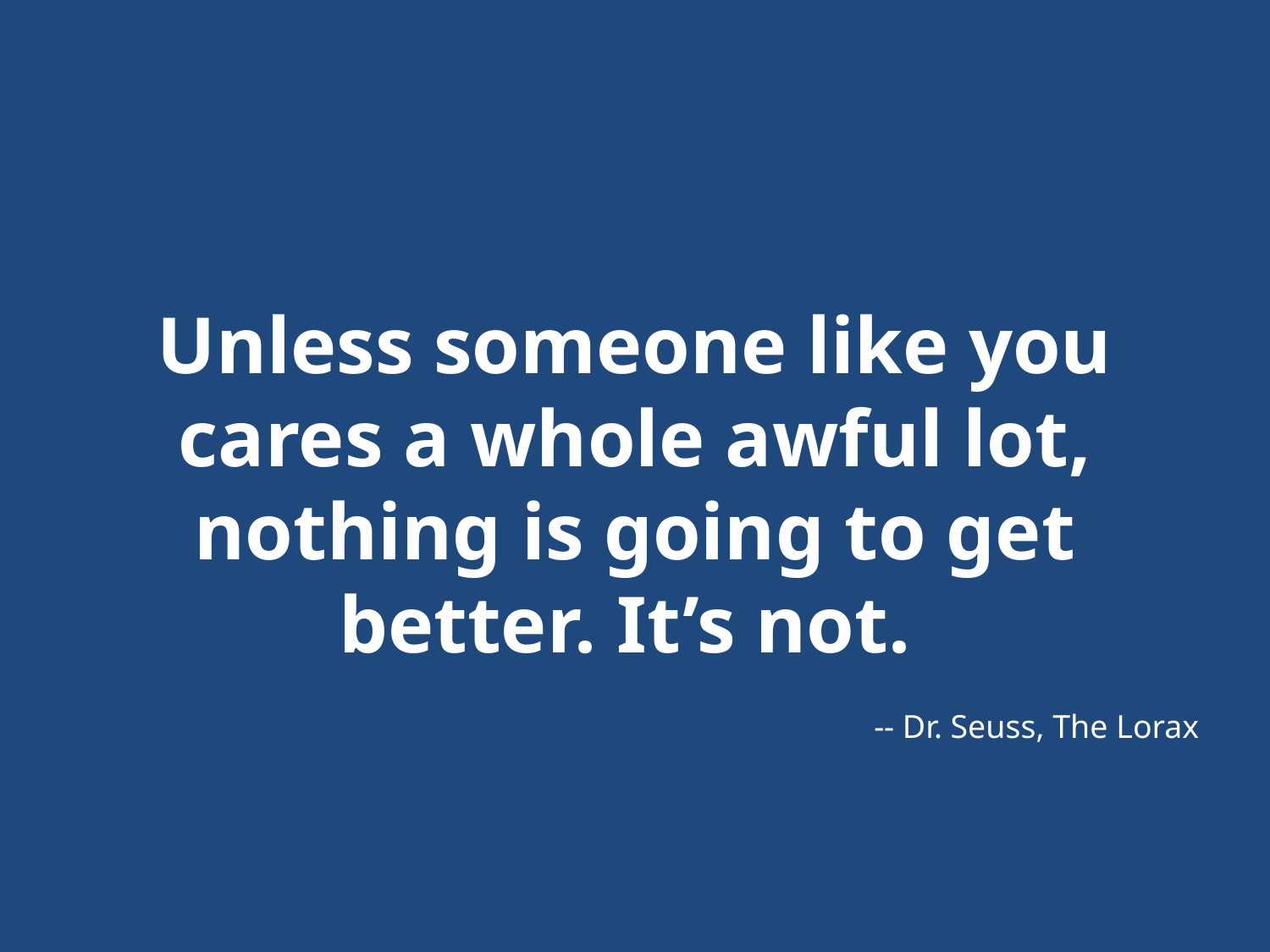

Unless someone like you cares a whole awful lot, nothing is going to get better. It’s not.
-- Dr. Seuss, The Lorax
