# Supplementary material for: Building an Antibiotic Stewardship Program: An Interactive Teaching Module for Medical Students
Source: MedEdPORTAL. 2018 Jun 26;14:10726. doi: 10.15766/mep_2374-8265.10726 (PMC6342413; doi:10.15766/mep_2374-8265.10726)
Supplement: Supplementary file 1 — A. ASP Presentation Slides.pptx B. Building an ASP Worksheet.docx C. One-Minute Paper.docx [file mep-14-10726-s001.zip › B._Building_an_ASP_Worksheet.docx]

**Building an Antibiotic Stewardship Program**

| Goals | The goal of this program is to |
| --- | --- |
| Objectives | 1.  2.  3. |
| Plan | Who: Consider the primary stakeholders.  Where: Consider the target clinical setting.  How: Consider the best or most high-yield interventions. |
| Resources & Logistics | Consider what resources/logistics you need to start the program and keep it running. |
| Potential Barriers | Delineate some barriers and how you might address them. |
| Outcomes | How will you measure your success? |
