# Supplementary material for: Building an Antibiotic Stewardship Program: An Interactive Teaching Module for Medical Students
Source: MedEdPORTAL. 2018 Jun 26;14:10726. doi: 10.15766/mep_2374-8265.10726 (PMC6342413; doi:10.15766/mep_2374-8265.10726)
Supplement: Supplementary file 1 — A. ASP Presentation Slides.pptx B. Building an ASP Worksheet.docx C. One-Minute Paper.docx [file mep-14-10726-s001.zip › C._One-Minute_Paper.docx]

**Regarding this session, please describe:**

1. A concept(s) you learned

2. A concept(s) you were unclear about

3. Something new you are going to try

Angelo TA, Cross, KP. Classroom assessment techniques: a handbook for college teachers, 2^nd^ ed. San Francisco, CA: Jossey-Bass; 1993.
